# New species and new records of camallanid nematodes (Nematoda, Camallanidae) from marine fishes and sea snakes in New Caledonia

**DOI:** 10.1051/parasite/2019068

**Published:** 2019-11-20

**Authors:** František Moravec, Jean-Lou Justine

**Affiliations:** 1 Institute of Parasitology, Biology Centre of the Czech Academy of Sciences Branišovská 31 370 05 České Budějovice Czech Republic; 2 Institut Systématique Évolution Biodiversité (ISYEB), Muséum National d’Histoire Naturelle, CNRS, Sorbonne Université, EPHE, Université des Antilles rue Cuvier, CP 51 75005 Paris France

**Keywords:** Nematoda, Helminth parasite, Camallanoidea, Aulopiformes, Carcharhiniformes, Perciformes, Pleuronectiformes, Serpentes, South Pacific

## Abstract

Recent examinations of camallanid nematodes (Camallanidae) from marine fishes off New Caledonia, collected in the years 2003–2011, revealed the presence of the following five new species of *Procamallanus* Baylis, 1923, all belonging to the subgenus *Spirocamallanus* Olsen, 1952: *Procamallanus* (*Spirocamallanus*) *dispar* n. sp. from the common ponyfish *Leiognathus equulus* (type host) and the striped ponyfish *Aurigequula fasciata* (both Leiognathidae, Perciformes); *Procamallanus* (*Spirocamallanus*) *bothi* n. sp. from the leopard flounder *Bothus pantherinus* (Bothidae, Pleuronectiformes); *Procamallanus* (*Spirocamallanus*) *hexophtalmatis* n. sp. from the speckled sandperch *Parapercis hexophtalma* (Pinguipedidae, Perciformes); *Procamallanus* (*Spirocamallanus*) *synodi* n. sp. from the sand lizardfish *Synodus dermatogenys* (Synodontidae, Aulopiformes); and *Procamallanus* (*Spirocamallanus*) *thalassomatis* n. sp. from the yellow-brown wrasse *Thalassoma lutescens* (Labridae, Perciformes). These are described based on light and scanning electron microscopical (SEM) studies. An additional three congeneric nematodes unidentifiable to species are reported from perciform fishes and a shark: *Procamallanus* (*Spirocamallanus*) sp. 3 of Moravec et al., 2006, *Procamallanus* (*Spirocamallanus*) sp. 1, and *Procamallanus* (*Spirocamallanus*) sp. 2. Ten fish species are recorded as new hosts for *Camallanus carangis* Olsen, 1954. Two camallanids, *Procamallanus* (*Spirocamallanus*) sp. 3 (subgravid female) and *Camallanus carangis* (fourth-stage larva) were also found in the digestive tract of the New Caledonian sea krait *Laticauda saintgironsi*, serving apparently as postcyclic and paratenic hosts, respectively, for these fish nematodes.

## Abstract (fr)

L’étude récente de nématodes camallanidés (Camallanidae) de poissons marins capturés au large de la Nouvelle-Calédonie, recueillis de 2003 à 2011, a révélé la présence des cinq nouvelles espèces suivantes de *Procamallanus* Baylis, 1923, toutes appartenant au sous-genre *Spirocamallanus* Olsen, 1952 : *Procamallanus (Spirocamallanus) dispar* n. sp. de *Leiognathus equulus* (hôte-type) et de *Aurigequula fasciata* (tous deux Leiognathidae, Perciformes) ; *Procamallanus* (*Spirocamallanus*) *bothi* n. sp. de *Bothus pantherinus* (Bothidae, Pleuronectiformes) ; *Procamallanus (Spirocamallanus) hexophtalmatis* n. sp. de *Parapercis hexophtalma* (Pinguipedidae, Perciformes) ; *Procamallanus (Spirocamallanus) synodi* n. sp. de *Synodus dermatogenys* (Synodontidae, Aulopiformes) ; et *Procamallanus (Spirocamallanus) thalassomatis* n. sp. de *Thalassoma lutescens* (Labridae, Perciformes). Ces espèces sont décrites sur la base d’études réalisées au microscope électronique à balayage et au microscope photonique. Trois autres nématodes congénères non identifiables au niveau spécifique sont signalés chez des poissons perciformes et un requin : *Procamallanus (Spirocamallanus)* sp. 3 de Moravec et al., 2006, *Procamallanus (Spirocamallanus)* sp. 1 et *Procamallanus (Spirocamallanus)* sp. 2. Dix espèces de poissons sont rapportées comme nouveaux hôtes pour *Camallanus carangis* Olsen, 1954. Deux camallanidés, *Procamallanus (Spirocamallanus)* sp. 3 (femelles subgravides) et *Camallanus carangis* (larve du quatrième stade) ont également été trouvés dans le tube digestif du serpent marin de Nouvelle-Calédonie, *Laticauda saintgironsi*, qui semble servir respectivement d’hôte post-cyclique et d’hôte paraténique pour ces nématodes de poissons.

## Introduction

Nematodes of the family Camallanidae Railliet et Henry, 1915, characterized by a well-developed, usually orange-coloured buccal capsule and a life cycle involving a copepod intermediate host, are mostly gastrointestinal, blood-sucking parasites of marine, brackish-water and freshwater fishes and, less often, of amphibians and aquatic reptiles (turtles, snakes) [1, 10, 41]. Although camallanids are frequent parasites of Indo-Pacific fishes, where many species have been reported, data on these nematodes in New Caledonian waters are scarce. To date, only the following nominal species of camallanids have been recorded from New Caledonia: *Camallanus cotti* Fujita, 1927 (an introduced species) and *Procamallanus* (*Procamallanus*) *pacificus* Moravec, Justine, Würtz, Taraschewski et Sasal, 2006 in freshwater fishes and *Camallanus carangis* Olsen, 1954, *Procamallanus* (*Procamallanus*) *annulatus* Yamaguti, 1955, *Procamallanus* (*Spirocamallanus*) *longus* Moravec, Justine et Rigby, 2006, *P*. (*S*.) *monotaxis* (Olsen, 1952), *P*. (*S*.) *sinespinis* Moravec et Justine, 2017 and *P*. (*S*.) *variolae* Moravec, Justine et Rigby, 2006 in marine fishes [12–14, 23–28].

Recent examinations of camallanid nematodes collected by J.-L. Justine and his students in marine fishes from off New Caledonia in the years 2003–2011 revealed the presence of five previously unknown species of *Procamallanus* Baylis, 1923 (subgenus *Spirocamallanus* Olsen, 1952) and three forms of the same subgenus unidentifiable to species, and showed several new hosts of one already known species of *Camallanus* Railliet et Henry, 1915. In addition, two fish camallanids were found in the digestive tract of sea snakes *Laticauda saintgironsi* Cogger et Heatwole. Results of this study are presented herein.

## Materials and methods

Fish were caught off New Caledonia by various means; those obtained from the fishmarket in Nouméa were very fresh and thus were probably fished in the near vicinity. For fish, we generally used the “wash” method [15]. For sea snakes, as these hosts are emblematic protected species, an indirect sampling method without any effect on the individual survival was used; a gentle massage of the sea krait abdomen provided the stomach content by regurgitation [4]. The regurgitated contents might include recently swallowed fish, which are thus recognizable [42], or, if digestion has already occurred, no recognizable item, as was the case for the samples described in this study. The nematodes for morphological studies were fixed in hot 4% formalin or 70% ethanol. For light microscopical examination, they were cleared with glycerine. Drawings were made with the aid of a Zeiss microscope drawing attachment. Specimens used for scanning electron microscopical (SEM) examination were postfixed in 1% osmium tetroxide (in phosphate buffer), dehydrated through a graded acetone series, critical-point-dried and sputter-coated with gold; they were examined using a JEOL JSM-7401F scanning electron microscope at an accelerating voltage of 4 kV (GB low mode). All measurements are in micrometres unless otherwise indicated. The fish nomenclature adopted follows FishBase [7].

## Results

### *Procamallanus (Spirocamallanus) dispar n.* sp. Figures 1, 2

urn:lsid:zoobank.org:act:579423E1-64EA-4D20-8511-64E5A5EB0753

**Figure 1:**
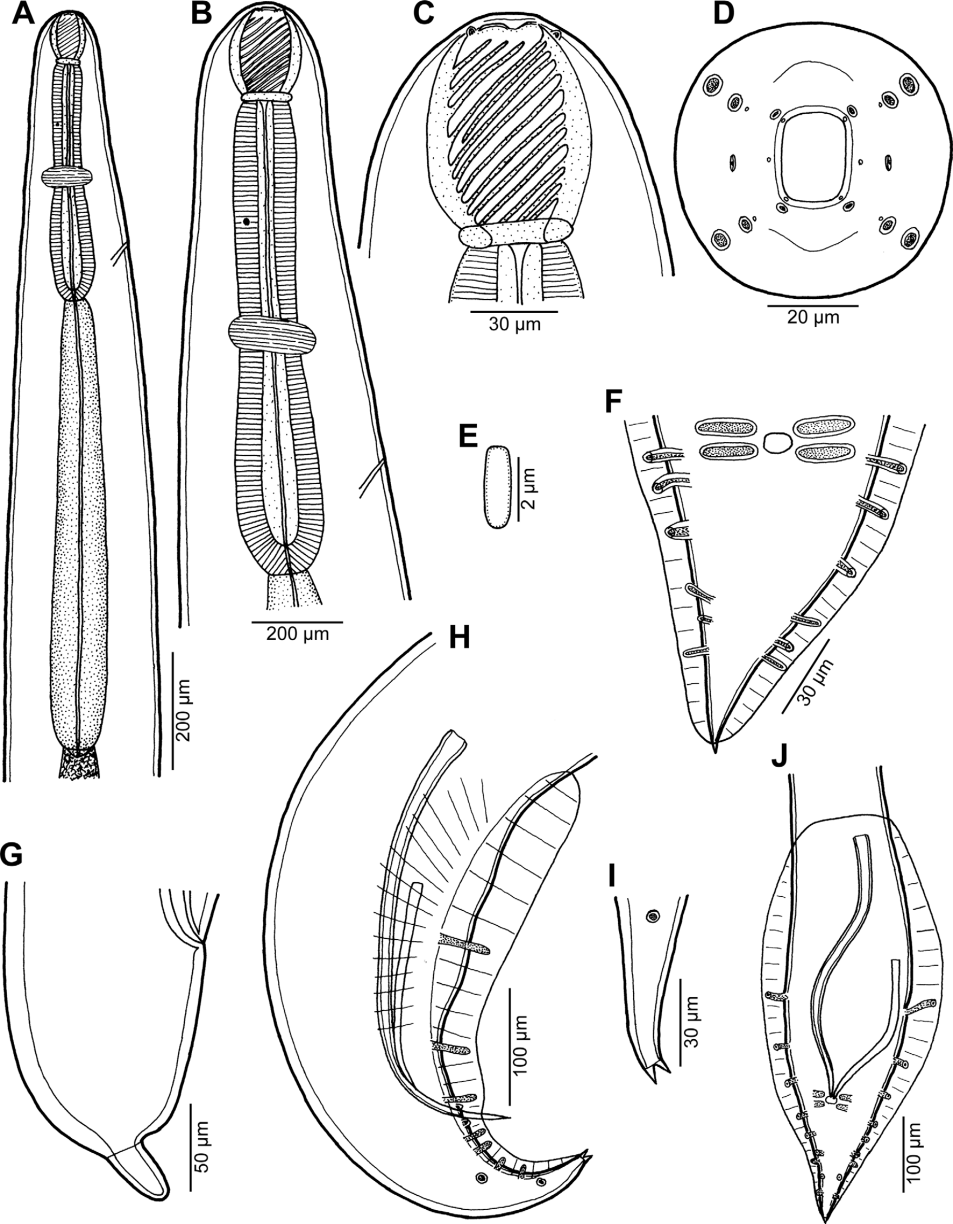
*Procamallanus* (*Spirocamallanus*) *dispar* n. sp. from *Leiognathus equulus*. (A) Anterior end of male, lateral view; (B) same, enlarged; (C, D) cephalic end of male, lateral and apical views, respectively; (E) deirid; (F) tail of male, ventral view; (G) tail of subgravid female, lateral view; (H) posterior end of male, lateral view; (I) tail tip of juvenile male, lateral view; (J) posterior end of juvenile male, ventral view.

**Figure 2:**
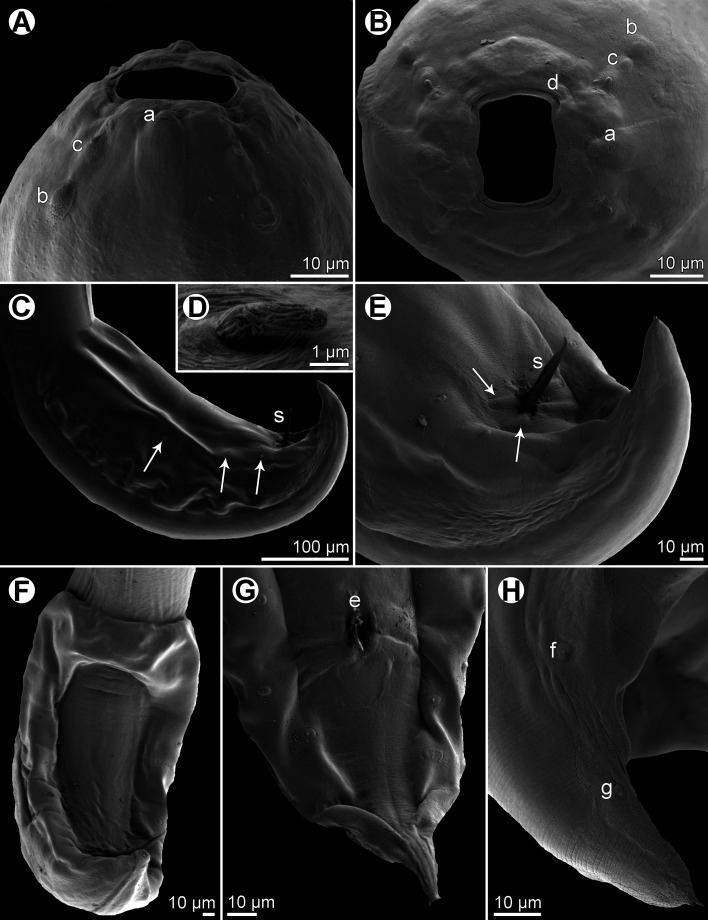
*Procamallanus* (*Spirocamallanus*) *dispar* n. sp., scanning electron micrographs of male. (A, B) Cephalic end, lateral and apical views, respectively; (C) posterior end of juvenile specimen, lateral view (arrows indicate subventral preanal papillae); (D) deirid; (E) tail, ventrolateral view (arrows indicate elongate papillae surrounding cloaca); (F) posterior end, ventral view; (G) tail of juvenile specimen, ventral view; (H) distal part of tail, lateral view. (a) amphid; (b) cephalic papilla of external circle; (c) cephalic papilla of middle circle; (d) cephalic papilla of internal circle; (e) cloacal aperture; (f) lateral postanal papilla; (g) phasmid; (s) spicule.

Type host: Common ponyfish *Leiognathus equulus* (Forsskål) (Leiognathidae, Perciformes).

Other host: Striped ponyfish *Aurigequula fasciata* (Lacepède) (Leiognathidae, Perciformes).

Site of infection: Intestine.

Type locality: Fishmarket, Nouméa, New Caledonia (collected 1 October 2008).

*Prevalence, intensity and details about fish*: *L*. *equulus*: 3 fish infected/4 fish examined; 2 nematodes per fish (Fish numbers: JNC2644, JNC2645, JNC2647, 1 October 2008). *A*. *fasciata*: 1 fish infected/4 fish examined; 1 nematode (Fish number: JNC2923, 29 April 2009). The infected fish were 129–137 mm in fork length and 62–68 g in weight (*L*. *equulus*), and 132 mm in length and 63 g in weight (*A*. *fasciata*).

Deposition of type specimens: Muséum National d’Histoire Naturelle, Paris, France (male holotype, female allotype and 1 paratype, MNHN JNC 2644 and JNC2647); Helminthological Collection, Institute of Parasitology, Biology Centre of the Czech Academy of Sciences, České Budějovice, Czech Republic (four male paratypes [three mounted on SEM stub], N–1200).

Etymology: The specific name of this nematode *dispar* (= different) is the Latin adjective relating to the fact that this species differs from related congeners.

#### Description

*General*: Medium-sized nematodes with finely transversely striated cuticle. Mouth aperture oval, surrounded by 12 submedian cephalic papillae arranged in three circles, each formed by four papillae; papillae of external circle distinctly larger; all papillae accompanied by distinct proximal pore; additional pair of pores situated laterally near edge of oral aperture; pair of small lateral amphids present (Figs. 1D, 2A and 2B). Buccal capsule orange, thick-walled, longer than wide, with simple, well-developed basal ring. Maximum width/length ratio of buccal capsule 1:1.12–1.39. Inner surface of capsule provided with 12–14 spiral ridges in lateral view, 4–6 of them being incomplete (Figs. 1B and 1C). Muscular oesophagus shorter than glandular oesophagus; both parts of oesophagus slightly expanded near their posterior ends (Figs. 1A and 1B). Intestine brown, narrow. Deirids small, simple, with rounded end situated approximately at mid-way between base of buccal capsule and nerve ring (Figs. 1A, 1B, 1E and 2D). Excretory pore located short distance anterior to posterior end of muscular oesophagus (Fig. 1B).

*Male* (four specimens, including holotype, from *L*. *equulus*; measurements of one juvenile specimen from *L*. *equulus* in parentheses. Measurements of one specimen from *A*. *fasciata* in brackets): Length of body 17.95–20.13 (10.47) [16.32] mm, maximum width 258–326 (218) [272]. Buccal capsule including basal ring 81–90 (84) [87] long, its width 66–75 (69) [72]; basal ring 9–12 (9) [9] long and 48–54 (45) [54] wide. Maximum width/length ratio of buccal capsule 1:1.12–1.36 (1:1.22) [1:1.21]. Spiral ridges 12–14 (16) [14], 6 (5) [5] of which incomplete. Length of muscular oesophagus 462–503 (435) [490], maximum width 69–87 (69) [87]; length of glandular oesophagus 734–884 (503) [653], maximum width 108–126 (84) [126]; length ratio of muscular and glandular oesophagus 1:1.59–1.85 (1:1.16) [1:1.33]. Length of entire oesophagus and buccal capsule representing 6–7 (10) [8]% of body length. Deirids, nerve ring and excretory pore 279–357 (297) [309], 358–326 (272) [299] and 394–462 (490) [–] from anterior extremity, respectively. Posterior end of body ventrally bent, provided with wide, vesiculated caudal alae supported by pedunculate papillae; anteriorly alae interconnected by mound, forming a kind of pseudosucker, and posteriorly reaching to caudal terminal spines (Figs. 1F, 1H–1J, 2C and 2E–2H). Preanal papillae: three pairs of subventral pedunculate papillae, of which second and third pairs closer to each other than first and second pairs; postanal papillae: six pairs of pedunculate papillae, four subventral and two lateral (last pair representing phasmids); additional two pairs of transversely-elongate sessile ventral papillae surrounding cloacal opening (Figs. 1F, 1H, 1J, 2E and 2G). Spicules unequal, with sharply pointed distal ends; large (right) spicule 426–449 (195) [408] long; small (left) spicule less sclerotized, 180–250 (90) [204] long. Length ratio of spicules 1:1.70–2.42 (1:2.17) [1:2.00]. Gubernaculum absent. Tail conical, 135–195 (138) [135] long, with two (dorsal and ventral) small terminal cuticular spines 3–6 (6) [6] long (Figs. 1F, 1H, 1I, 2G and 2H).

*Female* (one ovigerous specimen from *L*. *equulus*, allotype): Length of body 19.07 mm, maximum width 313. Buccal capsule including basal ring 96 long and 69 wide; basal ring 9 long and 57 wide. Maximum width/length ratio of buccal capsule 1:1.39. Number of spiral ridges 12, of which 4 incomplete. Length of muscular oesophagus 462, maximum width 87; length of glandular oesophagus 558, maximum width 114; length ratio of muscular and glandular oesophagus 1:1.21. Length of entire oesophagus and buccal capsule representing 6% of body length. Deirids, nerve ring and excretory pore 345, 272 and 452, respectively, from anterior extremity. Vulva postequatorial, 9.86 mm from anterior extremity, at 52% of body length. Vulval lips not elevated. Uterus filled with numerous eggs. Tail broad, rounded, its posterior end abruptly narrowed to form terminal digit-like protrusion with smooth, rounded tip; length of entire tail 162; digit-like protrusion 36 long, 18 wide (Fig. 1G).

#### Remarks

The present specimens from *L*. *equulus* and *A*. *fasciata* are considered to be conspecific because of their morphological and biometrical similarity and the fact that both of their host species belong to the same fish family. These nematodes belong to the subgenus *Spirocamallanus* of the genus *Procamallanus* in the conception of Moravec and Thatcher [32], namely to the group of *Spirocamallanus* species characterized by the presence of wide caudal alae, three pairs of pedunculate preanal papillae and two unequal spicules, that are mostly parasites of marine fishes [37]. Most species of this group are characterized by the presence of two caudal spikes, one dorsal and one ventral, on a digital projection in the female [9, 46], whereas these are lacking only in a few species. According to Petter et al. [38], Rigby and Adamson [39] and Moravec et al. [27], the shape and structure of the female tail appears to be constant within a species of *Procamallanus* (*Spirocamallanus*).

By the shape of the female tail and the absence of any terminal spikes, *P*. (*S*.) *dispar* n. sp. resembles only *P*. (*S*.) *mexicanus* Moravec, Salgado-Maldonado et Caspeta-Mandujano, 2000 from the freshwater cichlid *Cichlasoma geddesi* (Regan) (Cichlidae) in Mexico [29], *P*. (*S*.) *murrayensis* Johnston et Mawson, 1940 from freshwater perciform fishes *Pseudaphritis urvillii* (Valenciennes) (Pseudophritidae), *Macquaria colonorum* (Günther) and *M*. *ambigua* (Richardson) (both Percichthyidae) in Australia [11] and *P*. (*S*.) *sinespinis* from the marine fish *Pomadasys argenteus* (Forsskål) (Haemulidae) off New Caledonia [26]. Vicente and Santos [45] did not report the presence of two terminal caudal spines in females of the inadequately described *P*. (*S*.) *macaensis* Vicente et Santos, 1972, a parasite of several species of marine fishes in Brazil [16], but these are present according to the later redescription of this species [42].

However, in contrast to the new species, the right spicule of *P*. *murrayensis* is distinctly shorter (290 μm *vs* 408–449 μm). The right spicules of *P*. *mexicanus* and *P*. *sinespinis* are only slightly longer (456–480 μm and 465–525 μm, respectively *vs* 408–449 μm) than those of *P*. *dispar*, but the number of spiral ridges in their buccal capsules is 10–12 (*vs* 12–14); in addition, the male tail tip of these two species bears either a single conical cuticular spike (*P*. *mexicanus*) or a knob-like structure (*P*. *sinespinis*) (*vs* two, dorsal and ventral, terminal spikes are present); the female tail of *P*. *mexicanus* has a different shape, its anterior portion being narrow and conical (*vs* broad and posteriorly rounded). Moreover, *P*. *mexicanus* and *P*. *murrayensis* are parasites of freshwater fishes, whereas the hosts of *P*. *dispar* are marine fishes.

Moravec et al. [27] reported a nematode subgravid female, designated as *Procamallanus* (*Spirocamallanus*) sp. 3, from the marine fish *Scolopsis bilineata* (Bloch) (Nemipteridae) from off New Caledonia. The shape of its tail is similar to that of *P*. *dispar* and also the number (13) of spiral ridges in the buccal capsule corresponds to this species. However, the location of deirids is different (at the level of the nerve ring *vs* at the mid-way between the buccal capsule and the nerve ring), so that this specimen probably represents another species.

### *Procamallanus* (*Spirocamallanus*) *bothi* n. sp. Figures 3, 4

urn:lsid:zoobank.org:act:CCA32AF1-B9B7-4A1C-99FF-2C5A5A53410B

**Figure 3:**
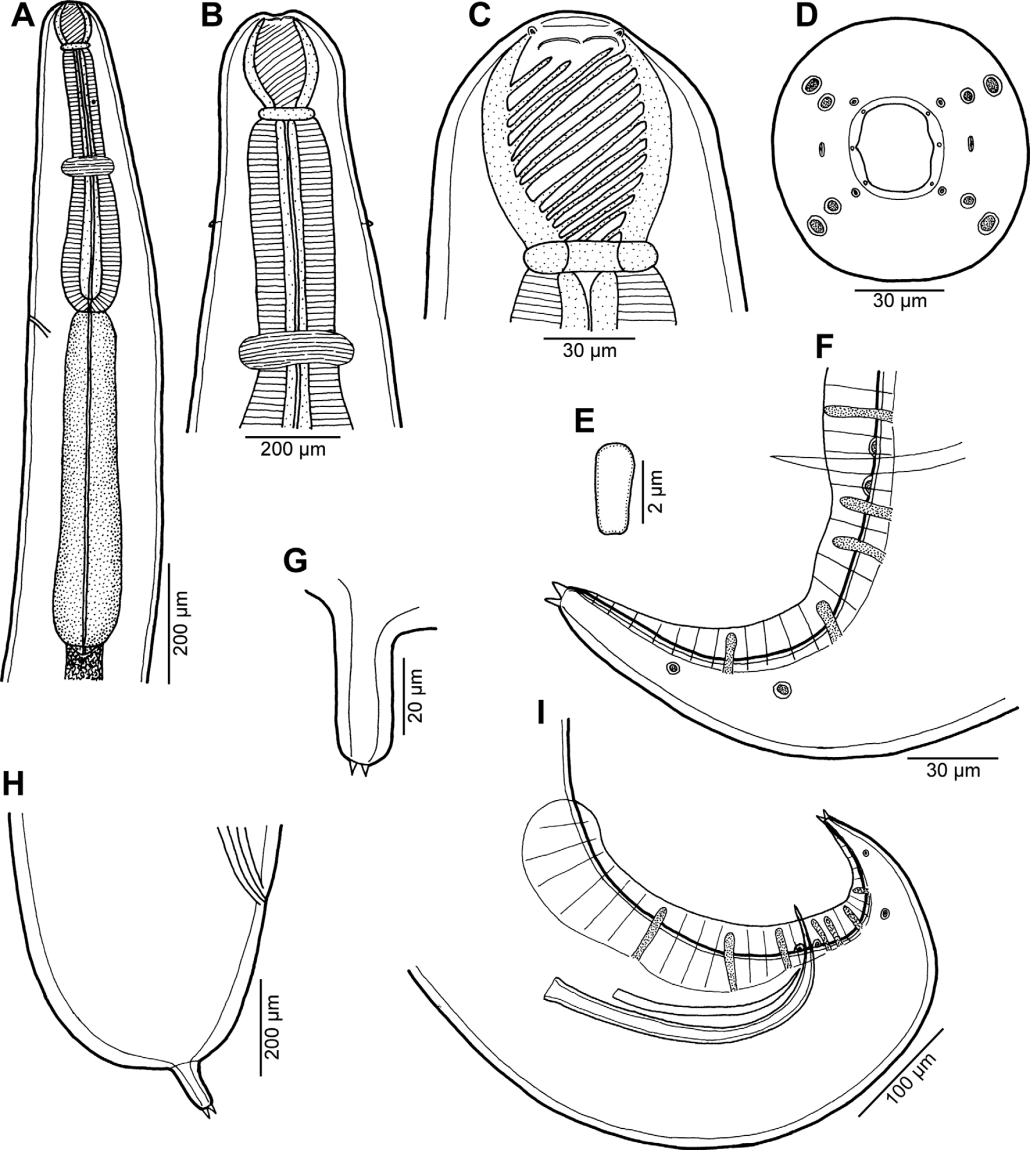
*Procamallanus* (*Spirocamallanus*) *bothi* n. sp. from *Bothus pantherinus*. (A, B) Anterior end of male, lateral and dorsoventral views, respectively; (C, D) cephalic end of male, lateral and apical views, respectively; (E) deirid; (F) tail of male, lateral view; (G) tail tip of female, lateral view; (H) tail of female, lateral view; (I) posterior end of male, lateral view.

**Figure 4:**
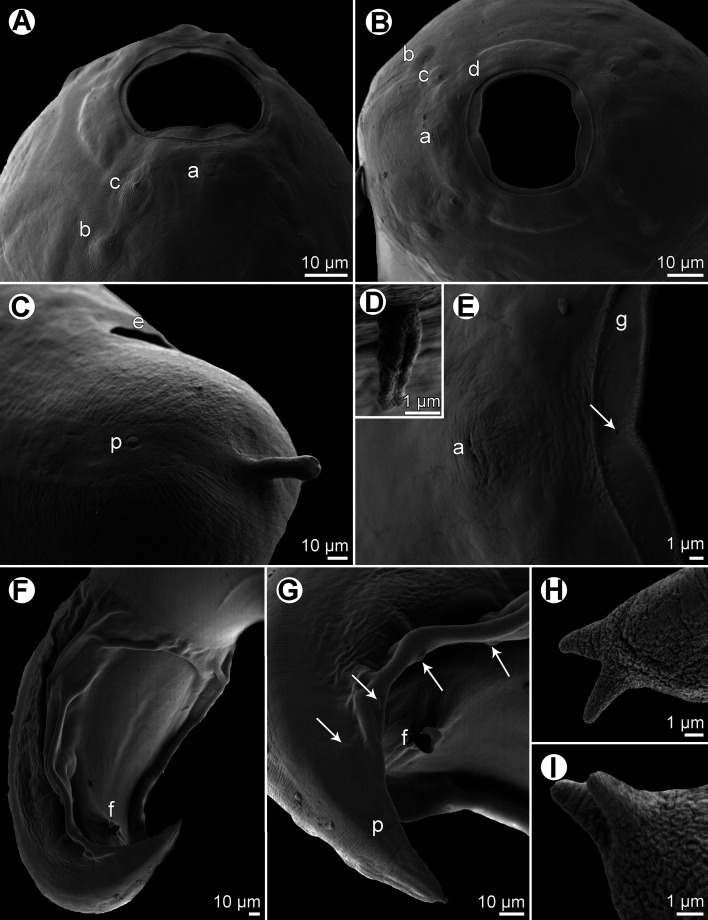
*Procamallanus* (*Spirocamallanus*) *bothi* n. sp., scanning electron micrographs. (A, B) Cephalic end, lateral and apical views, respectively; (C) female tail, sublateral view; (D) deirid; (E) region of amphid, apical view (arrow indicates lateral pore on margin of oral aperture); (F) posterior end of male, subventral view; (G) tail of male, ventrolateral view (arrows indicate pedunculated caudal papillae); (H) tail tip of male (lateral view); (I) tail tip of female, sublateral view). (a) amphid; (b) cephalic papilla of external circle; (c) cephalic papilla of middle circle; (d) cephalic papilla of internal circle; (e) anus; (f) cloacal aperture; (g) margin of oral aperture; (p) phasmid.

Type host: Leopard flounder *Bothus pantherinus* (Rüppell) (Bothidae, Pleuronectiformes).

Site of infection: Intestine.

Type locality: Fishmarket, Nouméa, New Caledonia (collected 11 February 2011).

*Prevalence, intensity and details about fish*: 1 fish infected/1 fish examined; 12 nematodes. The infected fish (Fish number: JNC3310) was 234 mm in fork length and 128 g in weight.

Deposition of type specimens: Muséum National d’Histoire Naturelle, Paris, France (male holotype, female allotype and eight paratypes, MNHN JNC 3310); Helminthological Collection, Institute of Parasitology, Biology Centre of the Czech Academy of Sciences, České Budějovice, Czech Republic (two paratypes mounted on SEM stub, N–1201).

Etymology: The specific name of this nematode relates to the genitive form of the generic name of the host.

#### Description

*General*: Medium-sized nematode with finely transversely striated cuticle. Mouth aperture oval, surrounded by 12 submedian cephalic papillae arranged in three circles, each formed by four papillae; papillae of outer circle larger; each of four small inner papillae present near margin of oral aperture accompanied by distinct proximal pore; pair of small lateral amphids present (Figs. 3D, 4A, 4B and 4E). Buccal capsule orange, thick-walled, longer than wide, with simple, well-developed basal ring. Maximum width/length ratio of buccal capsule 1:1.03–1.30. Inner surface of capsule provided with 13–19 spiral ridges in lateral view, 3–8 of them being incomplete (Figs. 3B and 3C). Muscular oesophagus shorter than glandular oesophagus; both parts of oesophagus slightly expanded near their posterior ends (Fig. 3A). Intestine brown, narrow. Deirids small, simple, with rounded end situated approximately at mid-way between base of buccal capsule and nerve ring (Figs. 3A, 3B, 3E and 4D). Excretory pore located short distance posterior to posterior end of muscular oesophagus (Fig. 3A).

*Male* (two specimens; holotype; measurements of paratype in parentheses): Length of body 18.20 (19.18) mm, maximum width 272 (367). Buccal capsule including basal ring 90 (90) long, its width 69 (72); basal ring 9 (9) long and 48 (51) wide. Maximum width/length ratio of buccal capsule 1:1.30 (1:1.25). Spiral ridges 14 (14), 6 (5) of which incomplete. Length of muscular oesophagus 476 (490), maximum width 102 (111); length of glandular oesophagus 612 (748), maximum width 123 (156); length ratio of muscular and glandular oesophagus 1:1.29 (1:1.53). Length of entire oesophagus and buccal capsule representing 6 (7)% of body length. Deirids, nerve ring and excretory pore 201 (195), 313 (313) and 639 (680) from anterior extremity, respectively. Posterior end of body ventrally bent, provided with wide, vesiculated caudal alae supported by pedunculate papillae; anteriorly alae interconnected by mound, forming a kind of pseudosucker, and posteriorly reaching to caudal terminal spines (Figs. 3F, 3I, 4F and 4G). Preanal papillae: three pairs of subventral pedunculate papillae, of which second and third pairs closer to each other than first and second pairs; postanal papillae: six pairs of pedunculate papillae, four subventral and two lateral (last pair representing phasmids); additional two pairs of small, transversely-elongate sessile ventral papillae surrounding cloacal opening (Figs. 3F, 3I and 4G). Spicules unequal, with sharply pointed distal ends; large (right) spicule 267 (270) long; small (left) spicule less sclerotized, 180 (177) long (Fig. 3I). Length ratio of spicules 1:1.48 (1:1.53). Gubernaculum absent. Tail conical, 207 (153) long, with two (dorsal and ventral) small terminal cuticular spines 6 (6) long (Figs. 3F, 3I and 4H).

*Female* (seven larvigerous specimens; measurements of allotype in parentheses. Measurements of additional three ovigerous specimens in brackets): Length of body 25.64–38.80 (37.92) [15.16–22.77] mm, maximum width 490–762 (734) [299–408]. Buccal capsule including basal ring 102–114 (102) [96–105] long and 84–105 (90) [84–99] wide; basal ring 9–12 (9) [9] long and 60–66 (60) [57–69] wide. Maximum width/length ratio of buccal capsule 1:1.06–1.27 (1:1.13) [1:1.03–1.25]. Number of spiral ridges 12–18 (18) [12–19], of which 3–5 (5) [3–8] incomplete. Length of muscular oesophagus 571–639 (598) [530–571], maximum width 129–136 (136) [69–111]; length of glandular oesophagus 721–911 (911) [612–748], maximum width 135–177 (177) [102–135]; length ratio of muscular and glandular oesophagus 1:1.22–1.52 (1:1.52) [1:1.15–1.38]. Length of entire oesophagus and buccal capsule representing 4–6 (4) [7–9]% of body length. Deirids, nerve ring and excretory pore 231–258 (233) [218–231], 340–381 (381) [313–354] and 707–884 (707) [639–680], respectively, from anterior extremity. Vulva mostly pre-equatorial (exceptionally equatorial), 11.37–18.90 (18.90) [7.71–10.36] mm from anterior extremity, at 36–50 (50) [46–50]% of body length. Vulval lips not elevated. Vagina directed posteriorly from vulva. Uterus filled with numerous larvae 390–404 long and 21–24 in maximum width, with slender tail [with small amount of eggs]. Tail broad, rounded, its posterior end abruptly narrowed to form digit-like protrusion provided with 2 (2) [2], dorsal and ventral, small terminal cuticular spikes; length of entire tail 190–218 (204) [138–204]; digit-like protrusion 36–51 (39) [21–42] long, 15–18) [12–15] wide, length of spines 3–5 (5) [5] (Figs. 3G, 3H, 4C and 4I).

#### Remarks

Nematodes of the present material belong to the morphological group of *Procamallanus* (*Spirocamallanus*) species characterized by the presence of wide caudal alae, three pairs of pedunculate preanal papillae, two unequal spicules and two caudal spikes on a digital projection in the female. According to Yooyen et al. [45], in the Indo-Pacific region this group contains 23 nominal species reported mostly from marine fishes. However, the great majority of them are poorly described and should be considered *species inquirendae* [see also 31, 40].

The following nine species of this morphological group from the Indo-Pacific region can be considered valid: *P*. (*S*.) *anguillae* Moravec, Taraschewski, Thairungroj Anantaphruti, Maipanich et Laoprasert, 2006 from *Anguilla bicolor* McClelland (Anguillidae) in Thailand and India [30, 31], *P*. (*S*.) *gobiomori* Moravec, Salgado-Maldonado et Caspeta-Mandujano, 2000 from freshwater Eleotridae in western Mexico [29], *P*. (*S*.) *guttatusi* (Andrade-Salas, Pineda-López et García-Magaña, 1994) from *Siganus guttatus* (Bloch) (Siganidae) off the Philippines [17, 22], *P*. (*S*.) *istiblenni* (Noble, 1966) from *Istiblennius zebra* (Vailland et Sauvage) (Blenniidae) off Hawaii [33, 40], *P*. (*S*.) *monotaxis* (Olsen, 1952) from Lethrinidae off Hawaii, New Caledonia and French Polynesia [25, 35, 39], *P*. (*S*.) *pereirai* (Annereaux, 1946) from *Atherinopsis californiensis* Girard (Atherinidae) off California, USA [3], *P*. (*S*.) *rigbyi* Yooyen, Moravec et Wongsawad, 2011 from *Otolithes ruber* (Bloch et Schneider) (Sciaenidae) off Thailand [46], *P*. (*S*.) *similis* Yooyen, Moravec et Wongsawad, 2011 from *Sillago sihama* (Forsskål) (Sillaginidae) off Thailand [46] and *P*. (*S*.) *variolae* Moravec, Justine et Rigby, 2006 from *Variola* spp. (Serranidae) off New Caledonia [27].

Of these, as compared with the new species, the right spicule is distinctly longer in *P*. *gobiomori* (318–348 μm *vs* 267–270 μm), *P*. *pereirai* (430 μm), *P*. *rigbyi* (315–360 μm), *P*. *similis* (435–492 μm) and *P*. *variolae* (327–357 μm); moreover, the spiral ridges in the buccal capsule are less numerous in *P*. *gobiomori* (8–10 *vs* 13–19), *P*. *similis* (10–12) and *P*. *variolae* (11–12) and all these five species also differ in the family and order of their fish hosts (Perciformes: Eleotridae, Serranidae, Sciaenidae and Sillaginidae or Atheriniformes: Atherinidae *vs* Pleuronectiformes: Bothidae). The right spicule of *P*. *anguillae* is somewhat longer (289–384 μm *vs* 267–270 μm) than that of *P*. *bothi* n. sp., the spiral ridges are usually less numerous (10–15 *vs* 13–19) and both species differ in the type of the host (freshwater eel *vs* marine flatfish). The length of the right spicule in *P*. *istiblenni* and *P*. *monotaxis* is rather similar to that in the new species (263–302 μm and 279–315 μm, respectively *vs* 267–270 μm), but the spiral ridges are mostly less numerous (12–15 and 10–17, respectively *vs* 13–19); deirids of *P*. *istiblenni* are located in 2/3 of the distance between the base of the buccal capsule and the nerve ring (*vs* in the mid-length of this distance) and the excretory pore at the level of the posterior end of the muscular oesophagus (*vs* somewhat posterior to this level), and the female tail of *P*. *istiblenni* is more conical as compared with that of *P*. *bothi* n. sp.; the excretory pore of *P*. *monotaxis* is located short distance anterior to the posterior margin of the muscular oesophagus (*vs* somewhat posterior to the anterior end of the glandular oesophagus). Moreover, the hosts of *P*. *istiblenni* and *P*. *monotaxis* belong to different fish families and orders (Perciformes: Blenniidae and Lethrinidae, respectively *vs* Pleuronectiformes: Bothidae).

### *Procamallanus* (*Spirocamallanus*) *hexophtalmatis* n. sp. Figures 5, 6

urn:lsid:zoobank.org:act:09400000-B876-4263-82A4-A0D2EA576751

**Figure 5:**
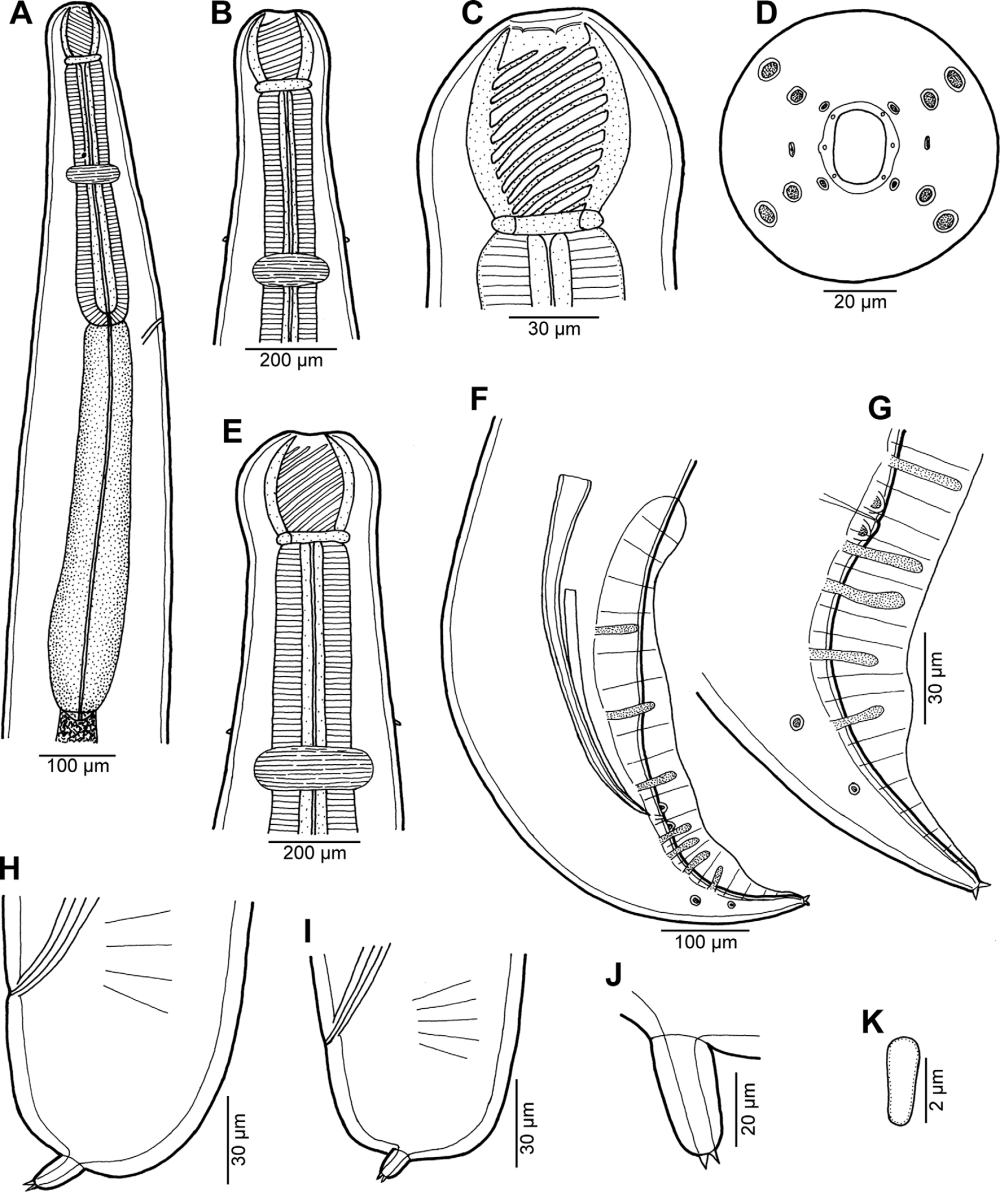
*Procamallanus* (*Spirocamallanus*) *hexophtalmatis* n. sp. from *Parapercis hexophtalma*. (A, B) Anterior end of male, lateral and dorsoventral views, respectively; (C, D) cephalic end of male, lateral and apical views, respectively; (E) anterior end of gravid female, dorsoventral view; (F) posterior end of male, lateral view; (G) tail of male, lateral view; (H, I) tail of gravid and subgravid female, respectively, lateral views; (J) tail tip of gravid female, lateral view; (K) deirid.

**Figure 6:**
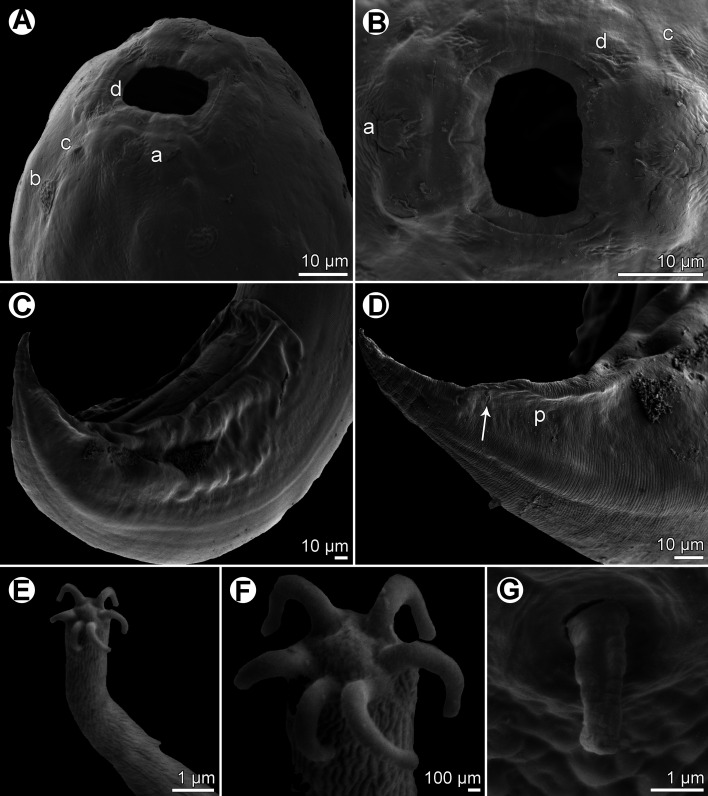
*Procamallanus* (*Spirocamallanus*) *hexophtalmatis* n. sp., scanning electron micrographs. (A, B) Cephalic end of female, sublateral and apical views, respectively; (C) posterior end of male, sublateral view; (D) posterior portion of male tail, lateral view (arrow indicates postanal papilla); (E) distal end of tail of first-stage larva; (F) digital appendages on tail tip of first-stage larva; (G) deirid. (a) amphid; (b) cephalic papilla of external circle; (c) cephalic papilla of middle circle; (d) cephalic papilla of internal circle; (p) phasmid.

Type host: Speckled sandperch *Parapercis hexophtalma* (Cuvier) (Pinguipedidae, Perciformes).

Site of infection: Intestine.

Type locality: Near Récif Toombo, off Nouméa, New Caledonia, 22°32′934 S, 166°28′549 E (collected 4 November 2008).

*Prevalence, intensity and details about fish*: 1 fish infected/2 fish examined; 4 nematodes (Fish number: JNC2736). The infected fish was 185 mm in fork length and 68 g in weight.

Deposition of type specimens: Helminthological Collection, Institute of Parasitology, Biology Centre of the Czech Academy of Sciences, České Budějovice, Czech Republic (male holotype and female allotype, both mounted on SEM stub, N–1202); Muséum National d’Histoire Naturelle, Paris, France (two paratypes, MNHN JNC 2736).

Etymology: The specific name of this nematode relates to the genitive form of the species name of the host.

#### Description

*General*: Medium-sized nematode with finely transversely striated cuticle. Mouth aperture oval, surrounded by 12 submedian cephalic papillae arranged in three circles, each formed by four papillae; papillae of outer circle distinctly larger; each of four small inner papillae present near margin of oral aperture accompanied by distinct proximal pore; pair of small lateral amphids present (Figs. 5D, 6A and 6B). Buccal capsule orange, thick-walled, longer than wide, with simple, well-developed basal ring. Maximum width/length ratio of buccal capsule 1:1.25–1.27. Inner surface of capsule provided with 11–12 spiral ridges in lateral view, of which three incomplete (Figs. 5B, 5C and 5E). Muscular oesophagus shorter than glandular oesophagus; both parts of oesophagus somewhat expanded near their posterior ends (Fig. 5A). Intestine brown, narrow. Deirids small, simple, with rounded end situated just anterior to level of nerve ring (Figs. 5A, 5B, 5E, 5K and 6G). Excretory pore located at level of junction of both parts of oesophagus (Fig. 5A).

*Male* (one complete specimen, holotype; measurements of one incomplete specimen in parentheses): Length of body 15.52 (body with missing posterior end 10.74) mm, maximum width 272 (231). Buccal capsule including basal ring 84 (75) long, its width 66 (60); basal ring 9 (6) long and 42 (39) wide. Maximum width/length ratio of buccal capsule 1:1.27 (1:1.25). Spiral ridges 12 (11) in number, 3 (3) incomplete. Length of muscular oesophagus 394 (354), maximum width 81 (69); length of glandular oesophagus 598 (558), maximum width 111 (72); length ratio of muscular and glandular oesophagus 1:1.52 (1:1.58). Length of entire oesophagus and buccal capsule representing 7 (–)% of body length. Deirids, nerve ring and excretory pore 222 (219), 258 (245) and 503 (435) from anterior extremity, respectively. Posterior end of body ventrally bent, provided with wide, vesiculated caudal alae supported by pedunculate papillae; anteriorly alae interconnected by mound, forming a kind of pseudosucker, and posteriorly reaching to caudal terminal spines (Figs. 5F, 5G, 6C and 6D). Preanal papillae: three pairs of subventral pedunculate papillae, of which second and third pairs closer to each other than first and second pairs; postanal papillae: six pairs of pedunculate papillae, four subventral and two lateral (last pair representing phasmids); additional two pairs of small, transversely-elongate sessile ventral papillae surrounding cloacal opening (Figs. 5F and 5G). Spicules unequal, with sharply pointed distal ends; large (right) spicule 324 (–) long; small (left) spicule less sclerotized, 180 (–) long (Fig. 5F). Length ratio of spicules 1:1.80 (–). Gubernaculum absent. Tail conical, 164 (–) long, with two (dorsal and ventral) small terminal cuticular spines 3 (–) long (Figs. 5F and 5G).

*Female* (one larvigerous specimen, allotype; measurements of one incomplete ovigerous specimen in parentheses): Length of body 24.00 (body with missing anterior end 14.31) mm, maximum width 571 (394). Buccal capsule including basal ring 99 (–) long and 78 (–) wide; basal ring 9 (–) long and 54 (–) wide. Maximum width/length ratio of buccal capsule 1:1.27 (–). Number of spiral ridges 11 (–). Length of muscular oesophagus 476 (–), maximum width 108 (–); length of glandular oesophagus 816 (–), maximum width 138 (–); length ratio of muscular and glandular oesophagus 1:1.71 (–). Length of entire oesophagus and buccal capsule representing 6 (–)% of body length. Deirids, nerve ring and excretory pore 299 (–), 313 (–) and 585 (–), respectively, from anterior extremity. Vulva pre-equatorial, 11.70 (–) mm from anterior extremity, at 49 (–)% of body length. Vulval lips not elevated. Vagina directed anteriorly from vulva. Uterus filled with numerous larvae 444–480 long and six in maximum width with slender tail (uterus with eggs); tail tip of larvae provided with six digital processes (Figs. 6E and 6F). Female tail broad, rounded, its posterior end abruptly narrowed to form digit-like protrusion provided with 2 (2), dorsal and ventral, small terminal cuticular spikes; length of entire tail 82 (58); digit-like protrusion 39 (21) long, 15 (12) wide, length of spines 3 (3) (Figs. 5H, 5I and 5J).

#### Remarks

As with the previous species, *P*. (*S*.) *hexophtalmatis* n. sp. also belongs to the morphological group of species of the subgenus *Spirocamallanus* characterized by the presence of wide caudal alae, three pairs of pedunculate preanal papillae, two unequal spicules and two caudal spikes on a digital projection in the female (see above). Of the species of this group occurring in the Indo-Pacific region, based on the length of the right spicule, *P*. *hexophtalmatis* n. sp. is similar to *P*. *anguillae* (289–384 μm *vs* 324 μm), *P*. *gobiomori* (318–348 μm), *P*. *guttatusi* (204–350 μm), *P*. *istiblenni* (263–302 μm), *P*. *monotaxis* (279–315 μm), *P*. *rigbyi* (315–360 μm) and *P*. *variolae* (327–357 μm) [17, 22, 25, 26, 29–31, 33, 35, 40, 46].

However, all these species, except for *P*. *variolae*, have deirids situated near the mid-point between the base of the buccal capsule and the nerve ring (*vs* deirids situated just anterior to the nerve ring). Based on this feature, the new species resembles only *P*. *variolae*, in which, however, the deirids are located slightly posterior (*vs* anterior) to the level of the nerve ring. Although *P*. *variolae* and *P*. *hexophtalmatis* n. sp. have the same numbers (11–12) of spiral ridges in the buccal capsule and the body lengths of their gravid (larvigerous) females are identical (approximately 24 mm), the new species differs from *P*. *variolae* in the length ratio of the muscular and glandular portions of the oesophagus (1:1.5–1.6 in males and 1:1.7 in the gravid female *vs* 1:1.1–1.3 in males and 1:1.3 in the gravid female), location of the excretory pore at the level of the muscular and glandular oesophageal junction (*vs* somewhat posterior to this junction) and in that the vagina of the gravid female is directed anteriorly (*vs* posteriorly) from the vulva. Whereas the tail of both gravid and subgravid females in the new species is widely rounded (Figs. 5H and 5I), that of the gravid female of *P*. *variolae* is somewhat more conical. Moreover, both these species differ in the family of their fish hosts (Pinguipedidae *vs* Serranidae).

In addition to a different location of deirids (see above), *P*. *anguillae*, *P*. *istiblenni*, *P*. *monotaxis* and *P*. *rigbyi* usually have more numerous spiral ridges in the buccal capsule (10–15, 12–15, 10–17 and 13–14, respectively *vs* 11–12), whereas the ridges of *P*. *gobiomori* are less numerous (8–10). These five species also differ from *P*. *hexophtalmatis* n. sp. in the family of their hosts (Anguillidae, Blenniidae, Lethrinidae, Sciaenidae and Eleotridae, respectively *vs* Pinguipedidae).

Moravec et al. [27] reported *Procamallanus* (*Spirocamallanus*) sp. 1 from *P*. *hexophtalma* in New Caledonia. However, although the general morphology of the only available specimen (subgravid female) was similar to that of *P*. *hexophtalmatis* n. sp., the spiral ridges in its buccal capsule were more numerous (16), deirids were located approximately at 2/3 of a distance between the base of the buccal capsule and the nerve ring and its tail was more conical as compared with that of the subgravid female of *P*. *hexophtalmatis*, resembling thus *P*. *monotaxis* [27]. Therefore, allocation of this specimen to *P*. *hexophtalmatis* is uncertain. Rigby and Adamson [39] reported *P*. *monotaxis*, originally described from a lethrinid fish from Hawaii [35], from *Parapercis millepunctata* (Günther) and members of several other fish families in French Polynesia, but this identification needs to be confirmed. In New Caledonia, *P*. *monotaxis* was recorded only from *Lethrinus* spp. [25].

The SEM examination of the first-stage larva of *P*. *hexophtalmatis* shows that the tail tip bears six digitiform processes (Figs. 6E and 6F). Similar caudal processes were previously observed in first-stage larvae of *Camallanus cotti* and *C*. *lacustris* [24] and those of two *Procamallanus* species from African freshwater fishes [18]. Apparently, these caudal processes serve the larva to better attach by its tail to the bottom, after the larvae are released into the water [24].

### *Procamallanus* (*Spirocamallanus*) *synodi* n. sp. Figures 7, 8

urn:lsid:zoobank.org:act:0F58977A-B15C-4888-ABD1-245B4016032E

**Figure 7:**
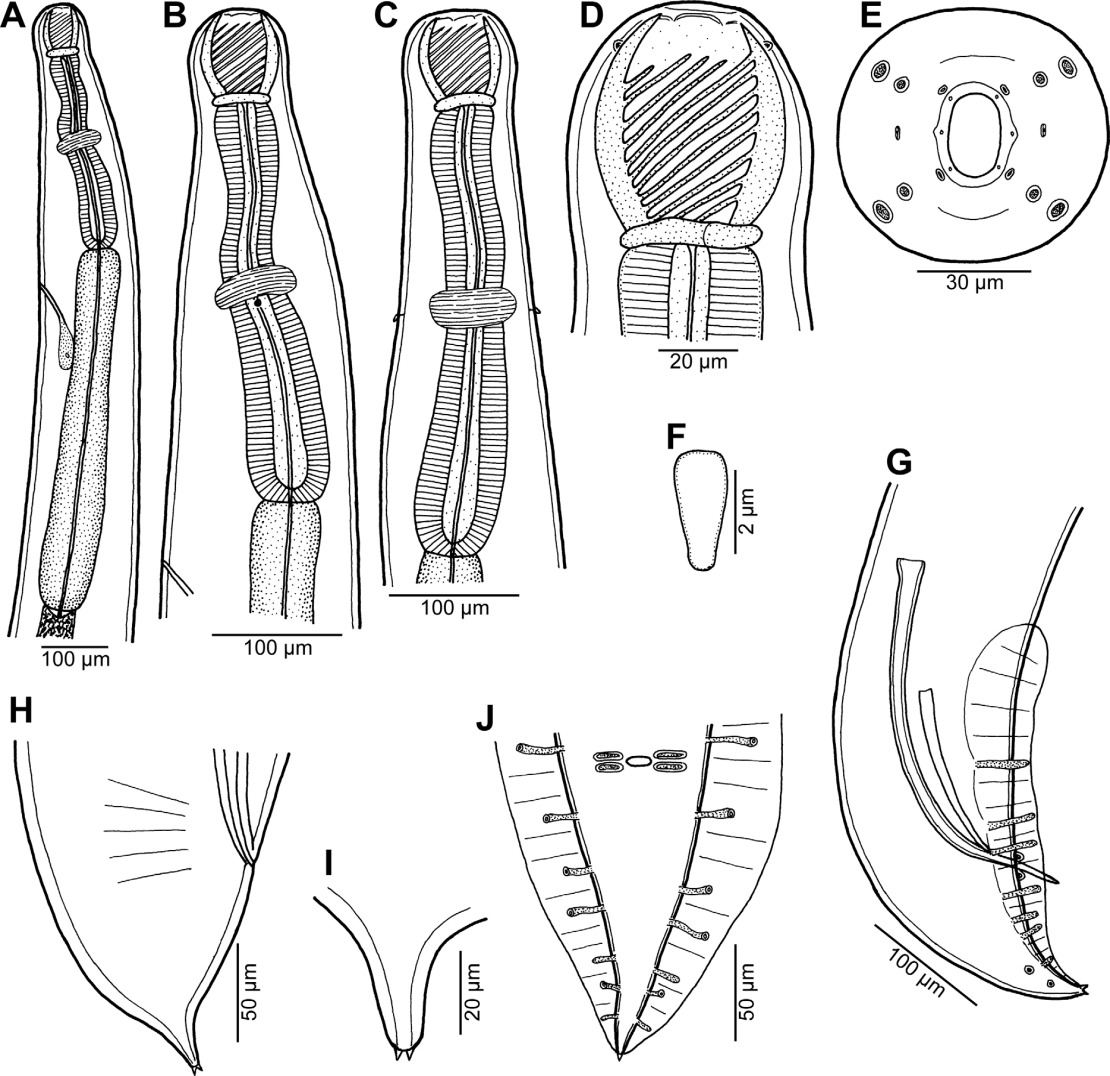
*Procamallanus* (*Spirocamallanus*) *synodi* n. sp. from *Synodus dermatogenys*. (A) Anterior end of male, lateral view; (B) same, larger magnification; (C) anterior end of female, dorsoventral view; (D) buccal capsule of male, lateral view; (E) cephalic end, apical view; (F) deirid; (G) posterior end of male, lateral view; (H) tail of female, lateral view; (I) tail tip of female, lateral view; (J) tail of male, ventral view.

**Figure 8:**
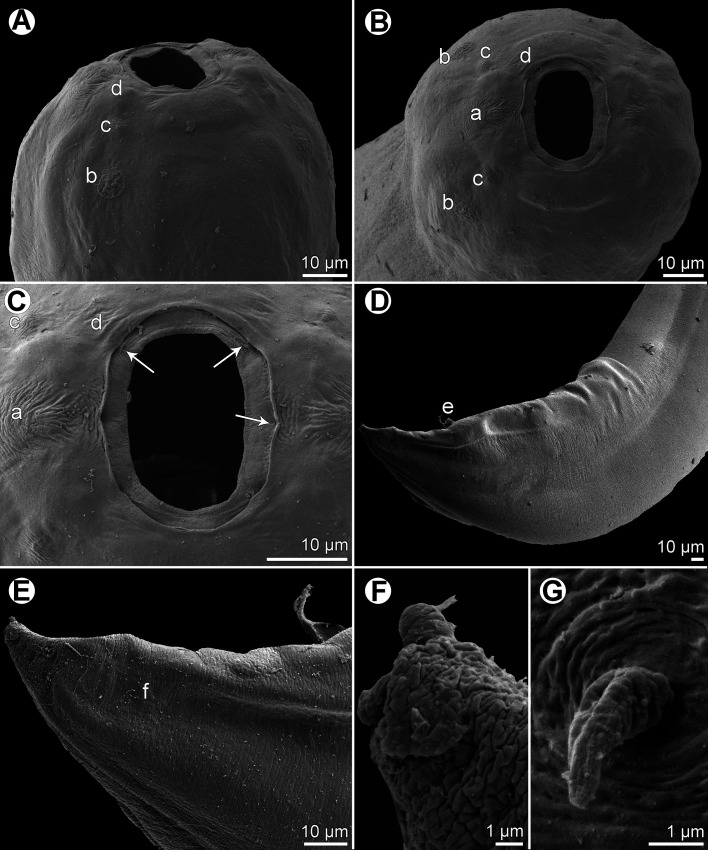
*Procamallanus* (*Spirocamallanus*) *synodi* n. sp., scanning electron micrographs. (A, B) Cephalic end, subdorsoventral and apical views, respectively; (C) region of oral aperture, apical view (arrows indicate circumoral pores); (D) posterior end of male, lateral view; (E) tail of male, lateral view; (F) tail tip of female, lateral view; (G) deirid. (a) amphid; (b) cephalic papilla of external circle; (c) cephalic papilla of middle circle; (d) cephalic papilla of internal circle; (e) cloaca; (f) phasmid.

Type host: Sand lizardfish *Synodus dermatogenys* Fowler (Synodontidae, Aulopiformes).

Site of infection: Intestine.

Type locality: Near Récif Toombo, off Nouméa, New Caledonia, 22°32′583 S, 166°28′978 E (collected 5 November 2008).

*Prevalence, intensity and details about fish*: 1 fish infected/4 fish examined; 5 nematodes. The infected fish (Fish number JNC2756) was 120 mm in fork length and 20 g in weight.

Deposition of type specimens: Helminthological Collection, Institute of Parasitology, Biology Centre of the Czech Academy of Sciences, České Budějovice, Czech Republic (male holotype and female allotype, both mounted on SEM stub, N–1203); Muséum National d’Histoire Naturelle, Paris, France (three paratypes, MNHN JNC 2756A).

Etymology: The specific name of this nematode relates to the genitive form of the generic name of the host.

#### Description

*General*: Medium-sized nematode with finely transversely striated cuticle. Mouth aperture oval, surrounded by 12 submedian cephalic papillae arranged in three circles, each formed by four papillae; papillae of outer circle distinctly larger; each of four small inner papillae present near margin of oral aperture accompanied by distinct proximal pore; pair of small lateral amphids present (Figs. 7E, 8A, 8B and 8C). Buccal capsule orange, thick-walled, longer than wide, with simple, well-developed basal ring. Maximum width/length ratio of buccal capsule 1:1.00–1.23. Inner surface of capsule provided with 10–12 spiral ridges in lateral view, 3–5 of them being incomplete (Figs. 7B, 7C and 7D). Muscular oesophagus shorter than glandular oesophagus; both parts of oesophagus slightly expanded near their posterior ends (Figs. 7A, 7B and 7C). Intestine brown, narrow. Deirids small, simple, with rounded end situated at level of nerve ring or just posterior to it (Figs. 7B, 7C, 7F and 8G). Excretory pore located short distance posterior to anterior end of glandular oesophagus (Fig. 7A).

*Male* (two specimens; holotype; measurements of paratype in parentheses): Length of body 9.38 (9.13) mm, maximum width 231 (218). Buccal capsule including basal ring 66 (81) long, its width 60 (66); basal ring 6 (9) long and 39 (48) wide. Maximum width/length ratio of buccal capsule 1:1.10 (1:1.23). Spiral ridges 12 (12), 0 (5) of which incomplete. Length of muscular oesophagus 354 (340), maximum width 63 (69); length of glandular oesophagus 462 (639), maximum width 78 (93); length ratio of muscular and glandular oesophagus 1:1.31 (1:1.88). Length of entire oesophagus and buccal capsule representing 9 (12)% of body length. Deirids, nerve ring and excretory pore 279 (246), 245 (231) and 462 (476), respectively, from anterior extremity. Posterior end of body ventrally bent, provided with wide, vesiculated caudal alae supported by pedunculate papillae; anteriorly alae interconnected by mound, forming a kind of pseudosucker, and posteriorly reaching to caudal terminal spines (Figs. 7G, 7J and 8D). Preanal papillae: three pairs of subventral pedunculate papillae, of which second and third pairs closer to each other than first and second pairs; postanal papillae: six pairs of pedunculate papillae, four subventral and two lateral (last pair representing phasmids); additional two pairs of small, transversely-elongate sessile ventral papillae surrounding cloacal opening (Figs. 7G, 7J and 8E). Spicules unequal, with sharply pointed distal ends (Fig. 7G); large (right) spicule 225 (330) long; small (left) spicule less sclerotized, 204 (147) long. Length ratio of spicules 1:1.10 (1:2.24). Gubernaculum absent. Tail conical, 123 (126) long, with two (dorsal and ventral) small terminal cuticular spines 3 (3) long (Fig. 7G).

*Female* (two ovigerous specimens; allotype; measurements of paratype in parentheses. Measurements of one nongravid specimen in brackets): Length of body 12.10 (11.74) [7.05] mm, maximum width 286 (218) [190]. Buccal capsule including basal ring 75 (66) [60] long and 72 (66) [51] wide; basal ring 9 (9) [6] long and 45 (39) [39] wide. Maximum width/length ratio of buccal capsule 1:1.04 (1:1.00) [1:1.18]. Number of spiral ridges 11 (11) [10], of which 4 (3) [3] incomplete. Length of muscular oesophagus 394 (422) [286], maximum width 75 (81) [54]; length of glandular oesophagus 530 (666) [394], maximum width 90 (111) [66]; length ratio of muscular and glandular oesophagus 1:1.35 (1:1.58) [1:1.38]. Length of entire oesophagus and buccal capsule representing 5 (10) [11]% of body length. Deirids, nerve ring and excretory pore 285 (285) [219], 245 (272) [190] and 544 (598) [408], respectively, from anterior extremity. Vulva equatorial or somewhat postequatorial, 6.03 (6.19) [3.86] mm from anterior extremity, at 50 (53) [55]% of body length. Vulval lips not elevated. Vagina directed posteriorly from vulva. Uterus filled with eggs (with eggs) [without eggs]. Tail broad, conical, its posterior end narrowed to form narrow conical protrusion provided with 2 (2) [2], dorsal and ventral, small terminal cuticular spikes; length of entire tail 171 (114) [96]; protrusion 24 (27) [45] long, 15 (15) [15] wide, length of spines 3 (3) [3] (Figs. 7H, 7I, 8F).

#### Remarks

The present nematodes belong to the same morphological group of *Procamallanus* (*Spirocamallanus*) as the previous two species, *P*. *bothi* n. sp. and *P*. *hexophtalmatis* n. sp. By the length of the right spicule, they resemble nine very similar species occurring in the Indo-Pacific region, viz. *P*. *anguillae*, *P*. *bothi* n. sp., *P*. *gobiomori*, *P*. *hexophtalmatis* n. sp., *P*. *guttatusi*, *P*. *istiblenni*, *P*. *monotaxis*, *P*. *rigbyi* and *P*. *variolae* (see above). However, in having deirids located at or near the level of the nerve ring, they resemble only *P*. *hexophtalmae* and *P*. *variolae*, whereas deirids in other species are situated approximately in the mid-point between the buccal capsule and the nerve ring (in *P*. *istiblenni* in 2/3 of this distance).

In contrast to the new species, the female tail of *P*. *hexophtalmatis* n. sp. is widely rounded (*vs* conical), deirids are located slightly anterior to the level of the nerve ring (*vs* deirids at or just posterior to this level), the excretory pore is at the level of the junction of both parts of the oesophagus (*vs* at a short distance posterior to the anterior end of the glandular oesophagus), the vulva is slightly pre-equatorial (*vs* equatorial or somewhat postequatorial), the vagina is directed anteriorly (*vs* posteriorly) from the vulva and the males and females are distinctly longer (male 15.5 mm, gravid female 24.0 mm *vs* males 9.1–9.4 mm, subgravid females 11.7–12.1 mm).

*Procamallanus* (*S*.) *variolae* differs from the new species in the shape of the female tail (rounded *vs* conical), in having a distinctly pre-equatorial vulva (*vs* vulva equatorial or postequatorial), a largely different length ratio of the muscular and glandular parts of the oesophagus (1:1.1–1.3 *vs* 1:1.3–1.9) and in that the males and females of this species are longer (males 10.5–12.7 mm, gravid female 24.5 mm *vs* males 9.1–9.4 mm and subgravid females 11.7–12.10 mm); the buccal capsule of *P*. *variolae* is larger (84–87 × 60–66 μm in males and 99 × 78 μm in female *vs* 66–81 μm in males and 66–72 × 66–72 μm in subgravid females). Moreover, the hosts of both *P*. *hexophtalmatis* and *P*. *variolae* belong to other fish families than that of the new species (Pinguipedidae and Serranidae, respectively *vs* Synodontidae).

*Procamallanus* (*S*.) *synodi* n. sp. is the first species of this genus reported from a fish of the family Synodontidae.

### *Procamallanus* (*Spirocamallanus*) *thalassomatis* n. sp. Figures 9–11

urn:lsid:zoobank.org:act:1B89617C-7328-471A-9A60-B25190D68E0E

**Figure 9:**
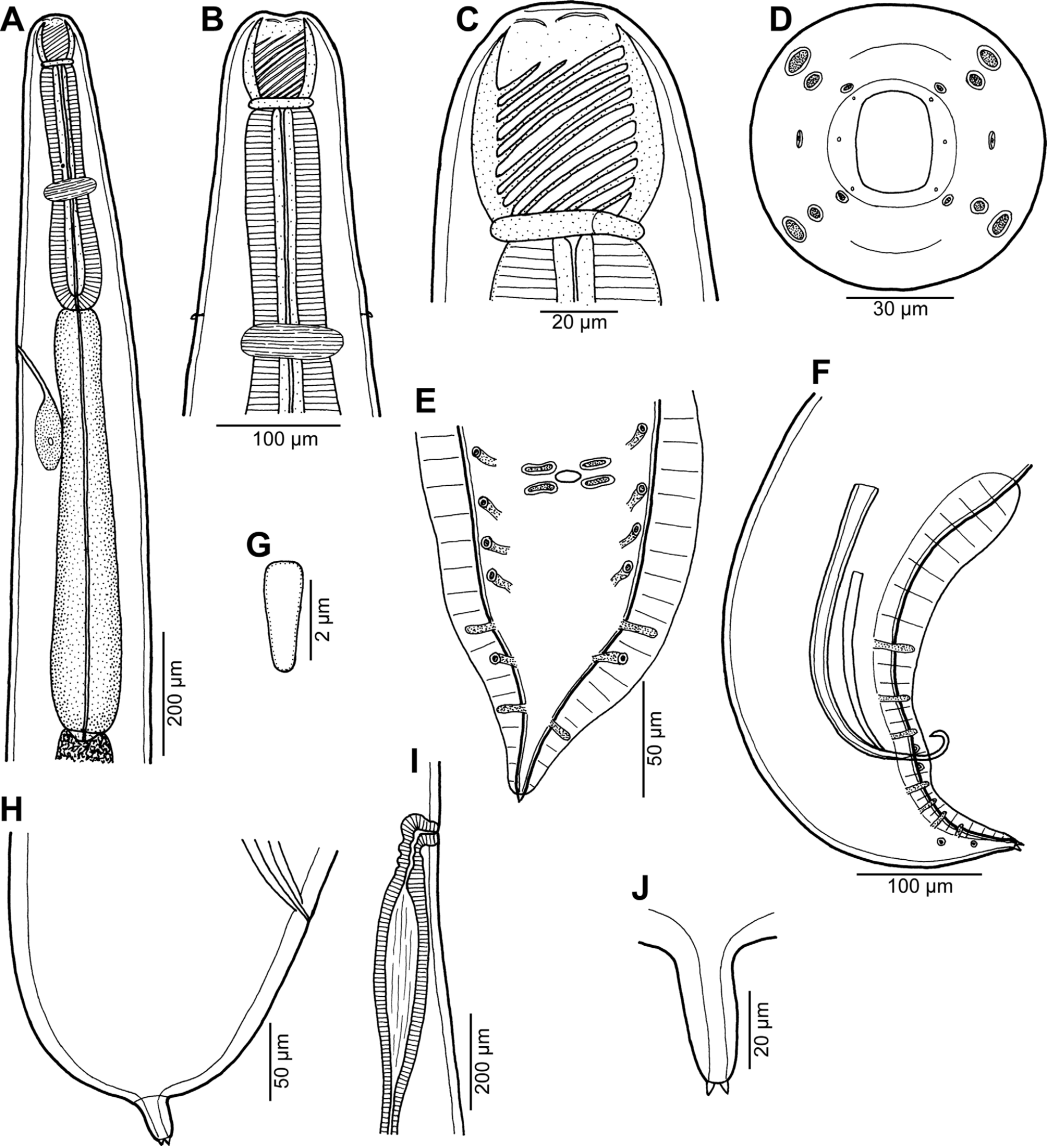
*Procamallanus* (*Spirocamallanus*) *thalassomatis* n. sp. from *Thalassoma lutescens*. (A, B) Anterior end of male, lateral and dorsoventral views, respectively; (C) buccal capsule of male, lateral view; (D) cephalic end, apical view; (E) tail of male, ventral view; (F) posterior end of male, lateral view; (G) deirid; (H) tail of female, lateral view; (I) vulva and distal end of vagina, lateral view; (J) tail tip of female, lateral view.

Type host: Yellow-brown wrasse *Thalassoma lutescens* (Lay et Bennett) (Labridae, Perciformes).

Site of infection: Intestine.

Type locality: Reef near Passe de Dumbéa, New Caledonia, 22°21′189 S, 166°15′158 E (collected 16 October 2009).

*Prevalence, intensity and details about fish*: 1 fish infected/8 fish examined; 3 nematodes. The infected fish (Fish number: JNC3074) was 173 mm in fork length and 63 g in weight.

Deposition of type specimens: Helminthological Collection, Institute of Parasitology, Biology Centre of the Czech Academy of Sciences, České Budějovice, Czech Republic (male holotype and female allotype, both mounted on SEM stub, N–1204); Muséum National d’Histoire Naturelle, Paris, France (female paratype, MNHN JNC 3074).

Etymology: The specific name of this nematode relates to the genitive form of the generic name of the host.

#### Description

**Figure 10:**
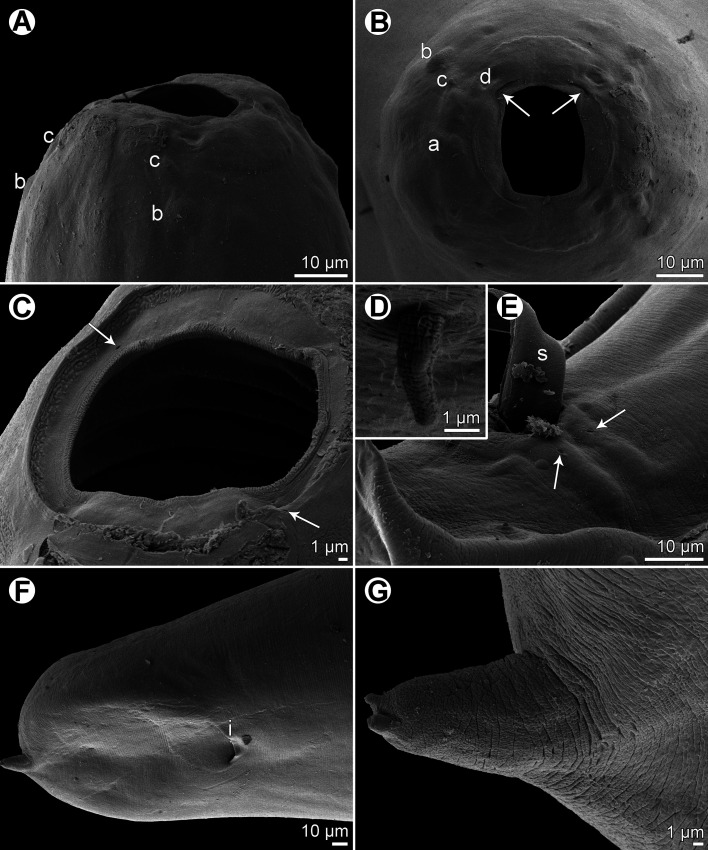
*Procamallanus* (*Spirocamallanus*) *thalassomatis* n. sp., scanning electron micrographs. (A, B) Cephalic end, sublateral and apical views, respectively (arrows indicate circumoral pores); (C) region of oral aperture, subapical view (arrows indicate circumoral pores); (D) deirid; (E) region of cloaca, sublateral view (arrows indicate pores of flat papillae around cloaca); (F) tail of gravid female, ventral view; (G) caudal projection of gravid female, sublateral view. (a) amphid; (b) cephalic papilla of external circle; (c) cephalic papilla of middle circle; (d) cephalic papilla of internal circle; (i) anus; (s) spicule.

*General*: Medium-sized nematode with finely transversely striated cuticle. Mouth aperture oval, surrounded by 12 submedian cephalic papillae arranged in three circles, each formed by four papillae; papillae of outer circle larger; each of four small inner papillae present near margin of oral aperture accompanied by distinct proximal pore; pair of small lateral amphids present (Figs. 9D, 10A, 10B and 10C). Buccal capsule orange, thick-walled, longer than wide, with simple, well-developed basal ring. Maximum width/length ratio of buccal capsule 1:1.07–1.21. Inner surface of capsule provided with 11–12 spiral ridges in lateral view, 4–5 of them being incomplete (Figs. 9B, 9C and 10C). Muscular oesophagus shorter than glandular oesophagus; both parts of oesophagus slightly expanded near their posterior ends (Fig. 9A). Intestine brown, narrow. Deirids small, simple, with rounded end situated slightly anterior to level of nerve ring (Figs. 9B, 9G and 10D). Excretory pore located somewhat posterior to anterior end of glandular oesophagus (Fig. 9A).

**Figure 11:**
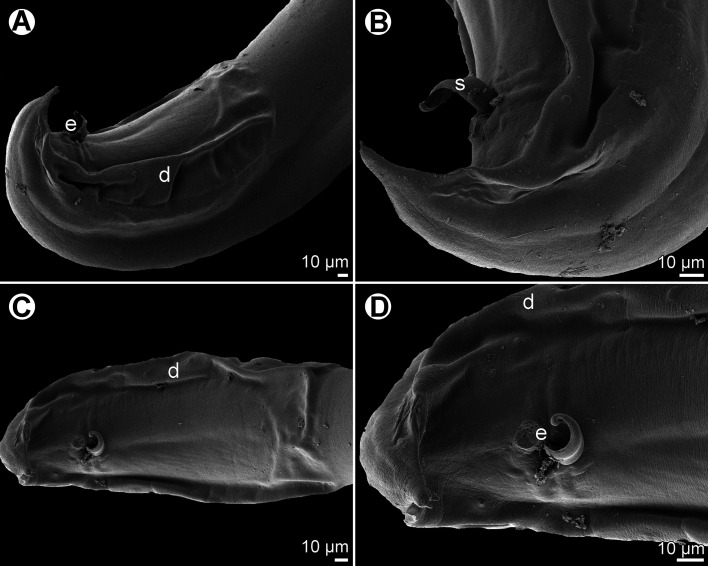
*Procamallanus* (*Spirocamallanus*) *thalassomatis* n. sp., scanning electron micrographs of male. (A) Posterior end of body, lateral view; (B) tail, lateral view; (C) posterior end of body, ventral view; (D) tail, ventral view. (d) caudal ala; (e) cloaca; (s) spicule.

*Male* (one specimen, holotype): Length of body 12.53 mm, maximum width 313. Buccal capsule including basal ring 87 long, its width 72; basal ring 12 long and 57 wide. Maximum width/length ratio of buccal capsule 1:1.21. Spiral ridges 12, 5 of which incomplete. Length of muscular oesophagus 435, maximum width 93; length of glandular oesophagus 748, maximum width 126; length ratio of muscular and glandular oesophagus 1:1.72. Length of entire oesophagus and buccal capsule representing 10% of body length. Deirids, nerve ring and excretory pore 279, 299 and 558, respectively, from anterior extremity. Posterior end of body ventrally bent, provided with wide, vesiculated caudal alae supported by pedunculate papillae; anteriorly alae interconnected by mound, forming a kind of pseudosucker, and posteriorly reaching to caudal terminal spines (Figs. 9E, 9F, 11A, 11B, 11C and 11D). Preanal papillae: three pairs of subventral pedunculate papillae, of which second and third pairs closer to each other than first and second pairs; postanal papillae: six pairs of pedunculate papillae, four subventral and two lateral (last pair representing phasmids); additional two pairs of small, transversely-elongate sessile ventral papillae surrounding cloacal opening (Figs. 9E, 9F, 10E, 11A, 11B and 11D). Spicules unequal, with sharply pointed distal ends (Fig. 9F); large (right) spicule 330 long; small (left) spicule less sclerotized, 144 long. Length ratio of spicules 1:1.48 (1:2.29). Gubernaculum absent. Tail conical, 141 long, with two (dorsal and ventral) small terminal cuticular spines four long (Figs. 9F and 11A).

*Female* (two larvigerous specimens; allotype; measurements of paratype in parentheses): Length of body 25.55 (25.66) mm, maximum width 639 (598). Buccal capsule including basal ring 96 (99) long and 90 (90) wide; basal ring 12 (12) long and 63 (63) wide. Maximum width/length ratio of buccal capsule 1:1.07 (1:1.10). Number of spiral ridges 11 (12), of which 4 (5) incomplete. Length of muscular oesophagus 625 (666), maximum width 136 (122); length of glandular oesophagus 1088 (1170), maximum width 177 (177); length ratio of muscular and glandular oesophagus 1:1.74 (1:1.76). Length of entire oesophagus and buccal capsule representing 7 (8)% of body length. Deirids, nerve ring and excretory pore 313 (326), 381 (367) and 762 (768), respectively, from anterior extremity. Vulva slightly pre-equatorial (equatorial), 12.65 (12.92) mm from anterior extremity, at 49 (50)% of body length. Vulval lips not elevated (Fig. 9I). Vagina directed posteriorly from vulva. Uterus filled with numerous larvae 381–396 long, with slender tail. Female tail broad, rounded, its posterior end abruptly narrowed to form digit-like protrusion provided with 2 (2), dorsal and ventral, small terminal cuticular spikes; length of entire tail 195 (201); digit-like protrusion 30 (42) long, 15 (15) wide, length of spines 3 (3) (Figs. 9H, 9J, 10F and 10G).

#### Remarks

The nematodes from *T*. *lutescens* belong to the same morphological group of *Procamallanus* (*Spirocamallanus*) as the species *P*. *bothi* n. sp., *P*. *hexophtalmatis* n. sp. and *P*. *synodi* n. sp. (see above). From the Indo-Pacific species of this group, *P*. *pereirai* and *P*. *similis* can be differentiated from *P*. *thalassomatis* n. sp. by possessing a distinctly longer right spicule (430 μm and 435–492 μm, respectively *vs* 330 μm) and *P*. *bothi* in having a shorter right spicule (267–270 μm), whereas the length of this spicule in the remaining species (*P*. *anguillae*, *P*. *gobiomori*, *P*. *guttatusi*, *P*. *istioblenni*, *P*. *monotaxis*, *P*. *rigbyi* and *P. variolae*) is rather similar. However, in having deirids located near the level of the nerve ring, they resemble only *P*. *hexophtalmatis*, *P*. *synodi* and *P*. *variolae*, whereas deirids in other species are situated approximately in the mid-way between the buccal capsule and the nerve ring (in *P*. *istiblenni* in 2/3 of this distance).

On the basis of the location of deirids somewhat anterior to the level of the nerve ring, *P*. *thalassomatis* n. sp. resembles *P*. *hexophtalmatis* n. sp., whereas deirids in *P*. *variolae* and *P*. *synodi* n. sp. are located at the level of the nerve ring or just posterior to this level. However, *P*. *thalassomatis* differs from *P*. *hexophtalmatis* in the vagina directed anteriorly (*vs* posteriorly) from the vulva; although the male body of the former species is shorter than that of the latter species (12.5 mm *vs* 15.5 mm), its buccal capsule is distinctly larger (87 × 72 μm *vs* 75–84 × 60 μm). The new species can be differentiated from *P*. *variolae* mainly by the length ratio of the muscular and glandular parts of the oesophagus (1:1.7–1.8 *vs* 1:1.1–1.3) and by the percentage of the length of the oesophagus and buccal capsule of the entire body length of gravid females (7–8% *vs* 5%), whereas from *P*. *synodi* mainly by the shape of the female tail (broadly rounded *vs* conical) and the larger buccal capsule (87 × 72 μm in male and 96–99 × 90 μm in gravid female *vs* 66–81 × 60–66 μm in male and 66–75 × 66–72 μm in subgravid female). Moreover, the hosts of *P*. *hexophtalmatis*, *P*. *synodi* and *P*. *variolae* belong to other fish families than that of the new species (Pinguipedidae, Synodontidae and Serranidae, respectively *vs* Labridae).

*Procamallanus* (*S*.) *thalassomatis* n. sp. is the first species of this genus reported from a fish of the family Labridae.

### *Procamallanus* (*Spirocamallanus*) sp. 3 of Moravec et al., 2006

Host: Two-lined monocle bream *Scolopsis bilineata* (Bloch) (Nemipteridae, Perciformes).

Site of infection: Intestine.

Locality: Near Ilôt Sainte-Marie, off Nouméa, New Caledonia, 22°18′S, 166°27′E (collected 4 July 2003 and 5 June 2006).

*Prevalence, intensity and details about fish*: 2 fish infected/12 fish examined; 1 nematode per fish. The infected fish (Fish numbers: JNC619, JNC1917) were 139–190 mm in fork length and 51–156 g in weight.

Deposition of voucher specimen: Muséum National d’Histoire Naturelle, Paris, France (1 specimen, MNHN JNC619A).

#### Remarks

Based on a single specimen (subgravid female) from *S*. *bilineata* off New Caledonia, Moravec et al. [27] described *Procamallanus* (*S*.) sp. 3, characterized by 13 spiral ridges in the buccal capsule and a broad tail with a short smooth projection. Two available specimens (also subgravid females) of the present material from the same host species, 14.12 and 18.84 mm long, are morphologically identical with that reported by Moravec et al. [27] and there is no doubt that both these forms belong to the same species. By the shape of the female tail and the absence of terminal spikes, this species is similar to *P*. (*S*.) *dispar* n. sp. and a few other congeners (see above). However, since conspecific males remain unknown, the species identification of these nematodes is impossible.

### *Procamallanus* (*Spirocamallanus*) sp. 1 Figure 12

Host: Shadow trevally *Carangoides dinema* Bleeker (Carangidae, Perciformes).

**Figure 12:**
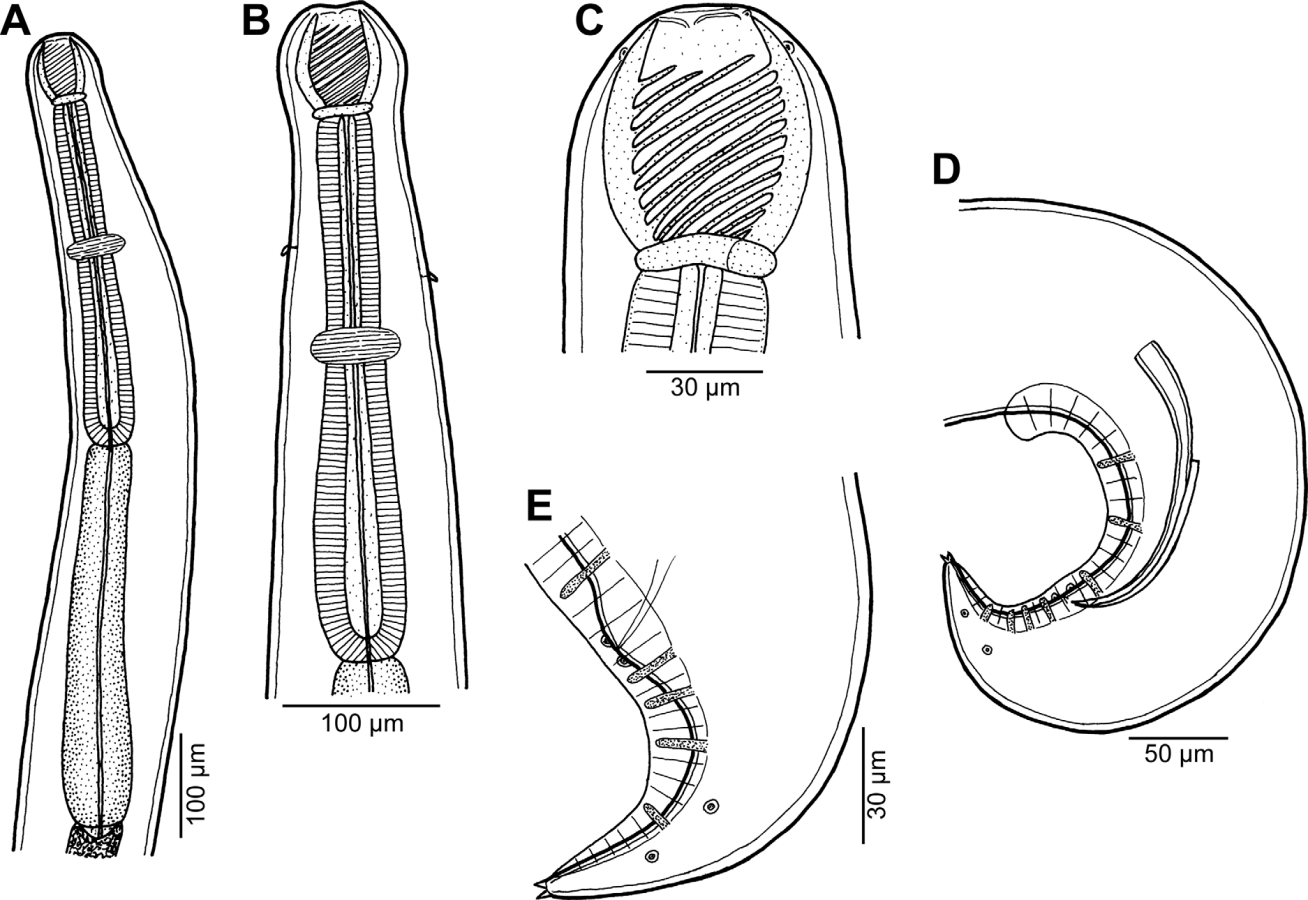
*Procamallanus* (*Spirocamallanus*) sp. 1 from *Carangoides dinema*, male. (A, B) Anterior end, lateral and dorsoventral views, respectively; (C) buccal capsule, lateral view; (D) posterior end of body, lateral view; (E) tail, lateral view.

Site of infection: Intestine.

Locality: Fishmarket, Nouméa, New Caledonia (collected 13 March 2009).

*Prevalence, intensity and details about fish*: 1 fish infected/9 fish examined; 1 nematode. The infected fish (Fish number: JNC2882) was 297 mm in fork length and 577 g in weight.

Deposition of voucher specimen: Muséum National d’Histoire Naturelle, Paris (MNHN JNC 2882).

#### Description

*Male* (one specimen): Length of body 7.56 mm, maximum width 163. Buccal capsule including basal ring 72 long, its width 60; basal ring 6 long and 39 wide. Maximum width/length ratio of buccal capsule 1:1.20. Spiral ridges 13, 5 of which incomplete (Fig. 12C). Length of muscular oesophagus 381, maximum width 60; length of glandular oesophagus 449, maximum width 81 (Figs. 12A and 12B); length ratio of muscular and glandular oesophagus 1:1.18. Length of entire oesophagus and buccal capsule representing 12% of body length. Nerve ring 231 from anterior extremity. Deirids situated somewhat asymmetrically at 165/195 from anterior extremity, slightly posterior to mid-point between base of buccal capsule and nerve ring (Fig. 12B). Excretory pore not located. Posterior end of body ventrally bent, provided with wide, vesiculated caudal alae supported by pedunculate papillae and posteriorly reaching to end of tail. Preanal papillae: three pairs of subventral pedunculate papillae; postanal papillae: six pairs of pedunculate papillae, four subventral and two lateral (last pair representing phasmids); additional two pairs of small, transversely-elongate sessile ventral papillae surrounding cloacal opening (Figs. 12D and 12E). Spicules unequal, with sharply pointed distal ends; right spicule 258 long; small (left) spicule less sclerotized, 198 long (Fig. 12D). Length ratio of spicules 1:1.30. Tail 114 long, with two (dorsal and ventral) small terminal cuticular spines three long (Fig. 12E).

#### Remarks

Only a single male specimen of this nematode was available to study. Because some taxonomically important morphological features are found in females in this group of nematodes (e.g., the shape of the female tail), species identification was not possible. No species of *Procamallanus* has so far been reported from a carangid fish.

### *Procamallanus* (*Spirocamallanus*) sp. 2 Figure 13

Host: Zebra shark *Stegostoma fasciatum* (Hermann) (Stegostomatidae, Orectolobiformes).

**Figure 13:**
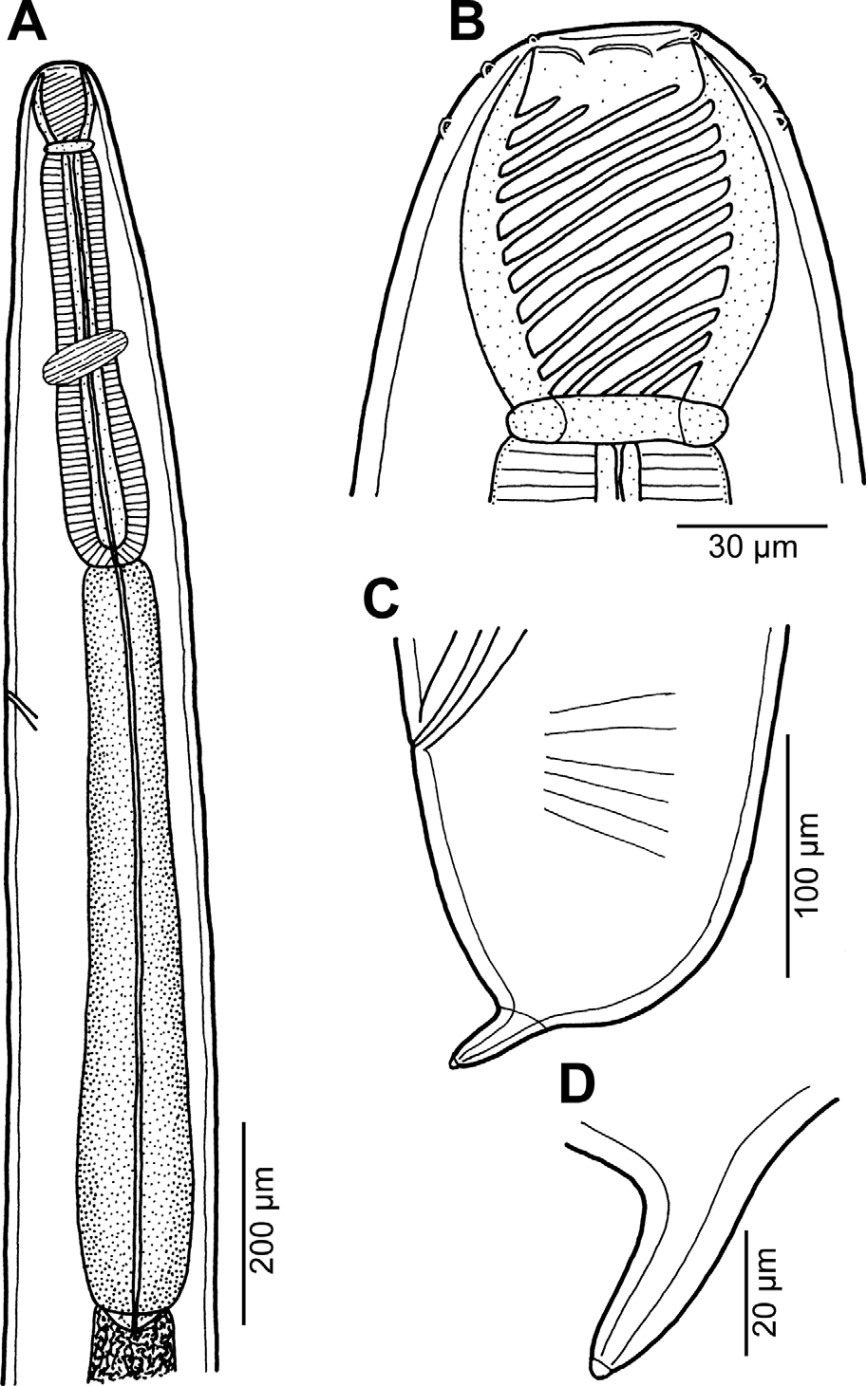
*Procamallanus* (*Spirocamallanus*) sp. 2 from *Stegostoma fasciatum*, subgravid female. (A) Anterior end, lateral view; (B) buccal capsule, lateral view; (C) tail, lateral view; (D) tail tip, lateral view.

Site of infection: Stomach.

Locality: Near Récif Aboré, off Nouméa, New Caledonia, 22°24′060 S, 166°18′961 E (collected 3 May 2005).

*Prevalence, intensity and detail about fish*: 1 fish infected/1 fish examined; 1 nematode; the shark was 2080 mm in length. Photograph of fish available from: https://commons.wikimedia.org/wiki/File:Stegostoma_fasciatum_JNC1529_Body.JPG

Deposition of voucher specimen: Muséum National d’Histoire Naturelle, Paris (MNHN JNC 1529).

#### Description

*Female* (one ovigerous specimen): Length of body 16.17 mm, maximum width 313. Buccal capsule including basal ring 93 long, its width 69; basal ring 9 long and 48 wide. Maximum width/length ratio of buccal capsule 1:1.35. Spiral ridges 12, of which 3 incomplete (Fig. 13B). Length of muscular oesophagus 490, maximum width 105; length of glandular oesophagus 843, maximum width 138 (Fig. 13A); length ratio of muscular and glandular oesophagus 1:1.18. Length of entire oesophagus and buccal capsule representing 12% of body length. Nerve ring 313 from anterior extremity. Deirids not located. Excretory pore at short distance posterior to posterior end of muscular oesophagus, at 734 from anterior end of body (Fig. 13A). Vulva postequatorial, 8.34 mm from anterior extremity, at 52% of body length. Vulval lips not elevated. Vagina directed posteriorly from vulva. Uterus filled with small amount of eggs. Tail broad, rounded, its posterior end abruptly narrowed to form digital protrusion provided with 1 small terminal cuticular knob; length of entire tail 163; digital protrusion 39 long, 15 wide (Figs. 13C and 13D).

#### Remarks

Due to availability of a single subgravid female, the species identification based on morphology is impossible. This species is characterized by the broadly rounded tail with a short projection without terminal cuticular spines. In this case, the shark apparently served as a postcyclic host, which had acquired the infection by feeding on the true definitive hosts (fish) of this nematode.

### *Procamallanus* (*Spirocamallanus*) sp. 3 Figure 14

Host: New Caledonian sea krait *Laticauda saintgironsi* Cogger et Heatwole (Elapidae, Serpentes).

**Figure 14:**
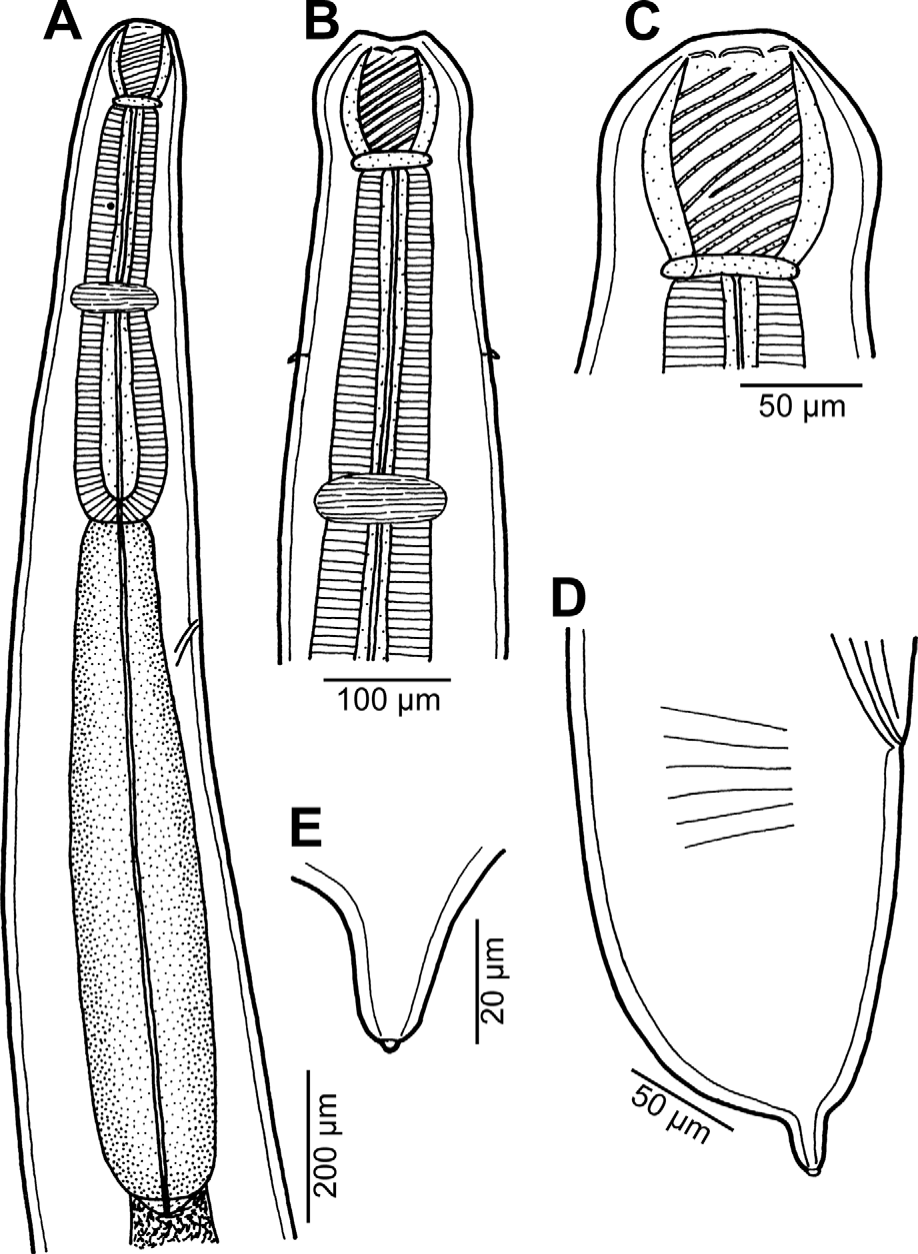
*Procamallanus* (*Spirocamallanus*) sp. 3 from *Laticauda saintgironsi*, subgravid female. (A, B) Anterior end, lateral and dorsoventral views, respectively; (C) buccal capsule, lateral view; (D) tail, lateral view; (E) tail tip, lateral view.

Site of infection: Collected from regurgitated digestive content of snake.

Locality: Île Amédée, off Nouméa, New Caledonia (collected 14 February 2011).

*Prevalence and intensity*: 1 snake infected/90 examined; 1 nematode.

Deposition of voucher specimen: Muséum National d’Histoire Naturelle, Paris, MNHN JNB011.

#### Description

*Female* (one ovigerous specimen): Length of body 23.11 mm, maximum width 462. Buccal capsule including basal ring 114 long, its width 90; basal ring 12 long and 66 wide. Maximum width/length ratio of buccal capsule 1:1.27. Spiral ridges 13, of which 5 incomplete (Fig. 14C). Length of muscular oesophagus 598, maximum width 105; length of glandular oesophagus 1020, maximum width 192 (Fig. 14A); length ratio of muscular and glandular oesophagus 1:1.71. Length of entire oesophagus and buccal capsule representing 7% of body length. Nerve ring 367 from anterior extremity. Deirids small, situated 243 from anterior extremity, approximately at mid-way between base of buccal capsule and nerve ring (Fig. 14B). Excretory pore located short distance posterior to posterior end of muscular oesophagus, at 816 from anterior end of body (Fig. 14A). Vulva postequatorial, 11.90 mm from anterior extremity, at 52% of body length. Vulval lips not elevated. Vagina directed posteriorly from vulva. Uterus filled with many eggs. Tail broad, somewhat conical, its posterior end abruptly narrowed to form digit-like protrusion provided with one small terminal cuticular knob; length of entire tail 122; digit-like protrusion 27 long, 15 wide (Figs. 14D and 14E).

#### Remarks

The presence of a single subgravid female but no male makes species identification of this nematode impossible. As in the previous case, it is apparent that the actual definitive host is a fish and the sea-snake acts only as a postcyclic host, which acquired the infection by feeding on fish. The nematode was collected from the regurgitated digestive content of a snake induced by manipulation, no identifiable prey fish was recovered.

### *Camallanus carangis* Olsen, 1954

Syns.: *Camallanus marinus* Schmidt et Kuntz, 1969; *C*. *paracarangis* Velasquez, 1980.

Hosts: Perciformes: Yellowtail scad *Atule mate* (Cuvier), yellowspotted trevally *Carangoides fulvoguttatus* (Forsskål), shadow trevally *Carangoides dinema* and bigeye scad *Selar crumenophthalmus* (Bloch) (all Carangidae), gold-saddle goatfish *Parupeneus cyclostomus* (Lacepède) and Indian goatfish *Parupeneus indicus* (Shaw) (both Mullidae), crimson jobfish *Pristipomoides filamentosus* (Valenciennes) (Lutjanidae), and tomato hind *Cephalopholis sonnerati* (Valenciennes), highfin grouper *Epinephelus maculatus* (Bloch), camouflage grouper *Epinephelus polyphekadion* (Bleeker) and red-tipped grouper *Epinephelus retouti* Bleeker (all Serranidae). Clupeiformes: dorab wolf-herring *Chirocentrus dorab* (Forsskål) (Chirocentridae). Gravid (larvigerous) females recorded only from *S*. *crumenophthalmus*. Only nematode fourth-stage larvae found in *P*. *indicus* and *C*. *dorab*.

In addition to fishes, a single fourth-stage larva of *C*. *carangis* was found in the regurgitated digestive content obtained from the New Caledonian sea krait *Laticauda saintgironsi* (Parasitological number JNB012) collected on Ilôt Signal, off Nouméa, on 28 February 2011.

Site of infection: Intestine.

Locality: Off Nouméa, New Caledonia.

*Prevalence, intensity and details about fish*: *Atule mate*: 1 fish infected/14 fish examined, 4 nematodes; infected fish, number JNC2963, 5 June 2009, Nouméa fishmarket, FL 318 mm, W 508 g. *Carangoides fulvoguttatus*: 1/15, 1 nematode; infected fish, number JNC3299, 28 January 2011, Nouméa fishmarket, FL 265 mm, W 409 g. *Carangoides dinema*: 1/9, 2 nematodes; infected fish, number JNC2882, 13 March 2009, Nouméa fishmarket, FL 293 mm, W 577 g. *Selar crumenophthalmus*: 2/10, 1–2 nematodes; infected fish, number JNC3043, 10 September 2009, Nouméa fishmarket, FL 227 mm, W 194 g and number JNC3125, Nouméa fishmarket, 25 November 2009, FL 240 mm, W 275 g. *Parupeneus cyclostomus*: 1/7, 2 nematodes; infected fish, number JNC1922, 22 August 2006, external reef near Ever Prosperity, off Nouméa, 22°27′28.8″ S, 166°21′50.4″ E, FL 263 mm, W 344 g; fish deposited in the ichthyological collections as MNHN-IC-2006-1753. *Parupeneus indicus*: 1/11, 1 nematode; infected fish, number JNC1964, 21 September 2006, Nouméa fishmarket, FL 290 mm, W 560 g. *Pristipomoides filamentosus*: 1/7, 1 nematode; infected fish, number JNC2460, 26 February 2008, near Passe de Dumbéa, off Nouméa, 22°22′ S, 166°14′ E, FL 360 mm, W 863 g. *Cephalopholis sonnerati*: 1/5, 3 nematodes; infected fish, number JNC2934, 12 May 2009, near Récif Toombo, off Nouméa, 22°31′40 S, 166°28′36 E, FL 474 mm, W 1600 g. *Epinephelus maculatus*: 1/38, 1 nematode; infected fish, number JNC2157, 17 April 2007, External reef near Ever Prosperity, off Nouméa, 22°27′28.8″ S, 166°21′50.4″ E, FL 535 mm, W 2050 g. *Epinephelus polyphekadion*: 1/3, 1 nematode; infected fish, number JNC3036, 8 September 2009, Passe de Dumbéa, off Nouméa, 22°21′232 S, 166°14′236 E, FL 410 mm, W 1055 g. *Epinephelus retouti*: 1/2, 2 nematodes; infected fish, number JNC2181, 19 June 2009, near Récif Toombo, off Nouméa, FL 329 mm, W 575 g. *Chirocentrus dorab*: 1/11, 1 nematode; infected fish, number JNC3236, 9 September 2010, Nouméa fishmarket, FL 700 mm, W 1538 g.

Deposition of voucher specimens: Muséum National d’Histoire Naturelle, Paris (MNHN JNC364B, JNC1903, JNC1922, JNC1964, JNC2157C, JNC2181, JNC2882, JNC2934, JNC2960, JNC2963, JNC3036, JNC3236, JNB013).

#### Remarks

The general morphology of the present specimens corresponds to that of *C*. *carangis*, as redescribed by Moravec et al. [27], and, therefore, they are assigned to this species. Since *C*. *carangis* has been redescribed and illustrated in detail by Moravec et al. [27], who also described its characteristic fourth-stage larva, we refrain from repeating the description of this species, based on newly collected specimens. Nevertheless, their measurements are compared in Tables 1–3.

**Table 1:** Comparison of measurements of *Camallanus carangis* males from marine fishes in New Caledonia.

Host	*Atule mate*	*Carangoides fulvoguttatus*	*Cephalopholis sonnerati*	*Epinephelus retouti*
No. of specimens	2	1	3	1
Body length (in mm)	12.17–13.67	6.90	9.93–11.32	frag. 5.40
Body width	299–354	218	258–326	258
Buccal capsule – length	165–189	162	183–186	159
Buccal capsule – width	159–189	156	156–183	165
No. of ridges	33–35	38	34–35	40
Basal ring – length	21–24	27	27–33	27
Basal ring – width	90–105	90	96–102	93
Length of tridents	150	150	171–219	153
Oesoph. cup – length	21–30	30	30–36	36
Oesoph. cup – width	24–33	39	36–39	30
Musc. oesoph. – length	1183–1414	1020	1333–1170	1102
Musc. oesoph. – width	122–136	81	122–136	84
Gland. oesoph. – length	1156–1333	857	1034–1238	1673
Gland. oesoph. – width	136	96	122–136	90
Musc./gland. oesoph. length ratio	1:0.94–0.98	1:084	1:0.88–0.93	1:0.79
% of buc. c. and oesoph. of body	21	30	23–24	–
Excretory pore	1047–1387	?	1115–1251	?
Right spicule	306–309	300	294–312	–
Tail	75–81	102	84–117	–

**Table 2:** Comparison of measurements of *Camallanus carangis* females from marine fishes in New Caledonia.

Host	*Atule mate*	*Carangoides dinema*	*Selar crumenophthalmus*	*Parupeneus cyclostomus*	*Pristipomoides filamentosus*	*Epinephelus retouti*
No. of specimens	1 (grav.)	2 (subgr.)	3 (gr., subgr.)	2 (subgr.)	1 (subgr.)	1 (subgr.)
Body length (in mm)	14.93	4.75 –7.00	14.63–17.00	10.23–11.13	14.89	10.95
Body width	340	163–245	381–517	340–408	313	354
Buccal capsule – length	195	159–180	186–225	159	150	183
Buccal capsule – width	198	144–180	204–233	144	159	195
No. of ridges	32	37–40	35–50	?	32	44
Basal ring – length	30	27	24–36	21–24	24	30
Basal ring – width	111	96	108–117	96–99	90	108
Length of tridents	141	135	195–240	105–195	141	153
Oesoph. cup – length	27	24–30	30–39	33	15	36
Oesoph. cup – width	36	30–36	39–45	36	33	30
Musc. oesoph. – length	1550	721–1047	1333–1850	816–979	911	1469
Musc. oesoph. – width	136	90	123–177	122–136	163	122
Gland. oesoph. – length	1333	653–911	1156–1659	775–925	993	1156
Gland. oesoph. – width	136	96–99	114–190	122–163	163	136
Musc./gland. oesoph. length ratio	1:0.86	1:0.87–0.91	1:0.87–0.98	1:0.94–0.95	1:1.09	1:0.79
% of buc. c. and oesoph. of body	21	22–40	18–25	17–19	14	26
Excretory pore	1401	707–911	1591	?	?	1224
Vulva from ant. end (in mm)	7.51	2.56–3.93	7.13–8.80	4.92	7.00	5.74
% of vulva of body	50	54–56	49–55	48	47	52
Tail	272	90–136	225	144–147	177	190

**Table 3:** Comparison of measurements of *Camallanus carangis* four-stage larvae from fish and reptilian hosts in New Caledonia.

Host	*Parupeneus indicus*	*Chirocentrus dorab*	Laticauda saintgironsi*
No. of specimens	1	1	1
Body length (in mm)	3.13	4.12	1.31
Body width	136	109	122
Buccal capsule – length	84	105	102
Buccal capsule – width	69	69	90
No. of ridges	17	?	18
Basal ring – length	12	21	24
Basal ring – width	51	51	54
Length of prongs	78	123	117
Oesoph. cup – length	18	24	18
Oesoph. cup – width	21	21	21
Musc. oesoph. – length	435	571	517
Musc. oesoph. – width	63	60	45
Gland. oesoph. – length	394	490	435
Gland. oesoph. – width	72	60	45
Musc./gland. oesoph. length ratio	1:091	1:0.86	1:0.84
% of buc. c. and oesoph. of body	2	28	81
Tail	72	147	54

* Sea-snake (Reptilia).

*Camallanus carangis*, originally described from *Caranx* sp. in Fiji [36], is known as a parasite of carangids and fishes belonging to some other families in Hawaii, French Polynesia, the Philippines, in the Arabian, Arafura, South China and Red Seas and also from off New Caledonia [23]. The present findings of *C*. *carangis* in fishes *Carangoides dinema*, *C*. *fulvoguttatus*, *Cephalopholis sonnerati*, *Chirocentrus dorab*, *Epinephelus maculatus*, *E*. *retouti*, *Parupeneus cyclostomus*, *P*. *indicus*, *Pristipomoides filamentosus* and *Selar crumenophthalmus*, as well as in the sea-snake *Laticauda saintgironsi*, represent new host records.

From New Caledonian waters, *C*. *carangis* was previously reported from marine perciform fishes *Carangoides chrysophrys* (Cuvier) and *C*. *hedlandensis* (Whitley) (Carangidae), *Nemipterus furcosus* (Valenciennes) (Nemipteridae), and *Parupeneus ciliatus* (Lacepède) and *Upeneus vittatus* (Forsskål) (both Mullidae) [23, 27]. The present survey extends considerably the range of hosts of *C*. *carangis* in New Caledonia, now including 15 fish species of the perciform families Carangidae, Lutjanidae, Mullidae, Nemipteridae and Serranidae, and a representative of the clupeiform family Chirocentridae. Of them, however, gravid (= larvigerous) females of this nematode have so far been recorded only from the carangid *S*. *crumenophthalmus* and the mullid *U*. *vittatus*, confirming thus that these fishes serve as the true definitive hosts for this parasite. Apparently, some of the hosts recorded serve only as paratenic, paradefinitive or postcyclic hosts [34], as is known, for example, for *Camallanus lacustris* (Zoega, 1776) or *C*. *oxycephalus* Ward et Magath, 1916, parasites of freshwater fishes in the Holarctic [20, 21, 44]. The present record of the *C*. *carangis* fourth-stage larva in the digestive tract of a sea-snake indicates that the snake acquired this infection while feeding on fish.

To date, *C*. *carangis* is the only representative of *Camallanus* parasitizing marine fishes in New Caledonian waters. Another congeneric species, *C*. *cotti*, a parasite of freshwater fishes, was introduced into New Caledonia [24].

## Discussion

All species of *Procamallanus* (*Spirocamallanus*) reported in this study belong to the morphological group of nematodes characterized by the presence of wide caudal alae, three pairs of pedunculate preanal papillae and two unequal spicules; as mentioned above, this mostly includes parasites of marine perciform fishes [37, 38]. Many species with these characteristics have been, often inadequately, described from different geographical zones, which makes a thorough comparison of them almost impossible. This situation is more complicated by the fact that some taxonomically important morphological features of these nematodes (e.g., the shape and position of deirids, excretory pore or the number and distribution of postanal papillae) are not easily observed under the light microscope and, consequently, that some insufficiently described species are reported from numerous, often unrelated hosts.

According to Petter et al. [38], Rigby and Adamson [39] and Moravec et al. [29], the shape and structure of the female tail of these nematodes appear to be constant within a species in *Procamallanus* (*Spirocamallanus*). Most species possess 2–4 terminal spines on the digital caudal projection in the female, whereas these are lacking only in a few species. Nevertheless, the morphology of all these species is rather similar. Although the division of *Procamallanus* (*Spirocamallanus*) species according to geographical zones by Andrade-Salas et al. [2] has been used by some authors [22, 27, 31, 39, 40] for the comparison of species, recent detailed morphological studies of some of these nematodes, including SEM, indicate a certain degree of their host specificity (approximately at the level of fish family), which should also be considered when evaluating these nematodes. This is supported by the present findings.

A quite different situation is regarding the species *C*. *carangis*, which, in New Caledonia, has been reported from 15 host species belonging to six fish families. This is not surprising, because a low degree of host specificity is well known for some other species of *Camallanus*, for example *C*. *cotti*, *C*. *lacustris*, *C*. *oxycephalus* or *C*. *truncatus* (Rudolphi, 1814). This is related with the circulation of these parasites in the environment, when different categories of hosts are employed during the development of these nematodes, i.e., different fishes may play a role of paratenic, definitive, paradefinitive or postcyclic hosts. Some aquatic snakes were found to be postcyclic hosts of the European species *C*. *lacustris* and *C*. *truncatus* [21].

Our records might be the first parasitological records for *L*. *saintgironsi*, a species recently described [6]; the species is endemic to New Caledonia [8] and sympatric with another species, *L*. *laticaudata* (Linnaeus) [5]. Its diet consists of non-spiny anguilliform fish, with the lipspot moray *Gymnothorax chilospilus* Bleeker representing about half of the prey [5]. The present records of *Procamallanus* (*S*.) sp. 3 subgravid female and that of *C*. *carangis* four-stage larva in the sea snakes *L*. *saintgironsi* indicate that these hosts acquired the infection with camallanids by feeding on fish hosts - probably morays - of these nematodes. Camallanids may survive in the digestive tract of fish-eating snakes for a long period (up to several months), as observed in *C*. *truncatus* overwintering in the European colubrid snake *Natrix tessellata* (Laurenti) [19]; in this case, it served as the postcyclic host. Regarding the above-mentioned camallanids in *L*. *saintgironsi*, these snakes served as postcyclic and paratenic hosts, respectively.
